# Investigation of the synergistic effects of UV radiation and elevated temperatures on regenerated cellulose fiber-reinforced bio-polyamide 5.10 composites

**DOI:** 10.1038/s41598-026-51172-z

**Published:** 2026-04-29

**Authors:** Celia Katharina Falkenreck, Jan-Christoph Zarges, Hans-Peter Heim

**Affiliations:** Institute of Materials Engineering, Plastics Engineering, University of Kassel, Moenchebergstr. 3, 34125 Kassel, Germany

**Keywords:** Regenerated cellulose fibers, Bio-polyamide, Degradation, UV-radiation, Thermo-oxidation, Photo-oxidation, Chemistry, Engineering, Materials science

## Abstract

This study examines the thermo- and photo-oxidative degradation of bio-based polyamide 5.10, both neat and reinforced with 20 wt% regenerated cellulose fibers (RCF). The materials were additionally modified with two UV stabilizers: a hindered amine light stabilizer (HALS) and a UV absorber. Samples were stored for 168 h at 23 °C, 50 °C, 70 °C, and 90 °C under 50% rH, with and without 1000 W/m^2^ UV exposure. Pronounced degradation was observed in the neat and non-stabilized batches, including molecular chain splitting, embrittlement, surface polarity reduction, and yellowing. In contrast, HALS-containing formulations exhibited superior stabilization, retaining thermal, mechanical, and optical properties. RCF-reinforced PA5.10 showed moisture-induced plasticization at moderate conditions and embrittlement at higher temperatures. SEM analysis revealed increased fiber ruptures in UV-aged neat composites, whereas HALS-stabilized specimens maintained predominant fiber pull-out behavior. Melt volume rate testing confirmed molecular weight reduction due to oxidative degradation and a linear polynomial regression was used to demonstrate the characteristic degradation mechanisms. Overall, HALS provided effective protection against both thermal and photo-chemical aging across all storage conditions. These findings demonstrate the potential of HALS-stabilized, RCF-reinforced PA5.10 composites as sustainable alternatives to petro-chemical polyamides for applications demanding long-term resistance to combined thermo- and photo-oxidative stress.

## Introduction

With increasing focus on sustainability in materials engineering, natural fiber composites (NFC) have emerged as eco-friendly alternatives to glass fiber composites (GFC), offering advantages in weight, cost, and environmental impact^1–3^. However, natural fibers often face limitations such as thermal instability, inconsistent mechanical properties, and hygroscopic behavior^4,5^. Regenerated cellulose fibers (RCF), such as viscose, present a promising reinforcement due to their improved thermal resistance and reduced variability compared to plant-based fibers^3^. Their ductile failure behavior and lower impurity content make them particularly attractive for industrial applications. To develop bio-based composites, an appropriate bio-based matrix material is required. Compared to other bio-based plastics, such as PLA, bio-based polyamides (e.g., PA10.10, PA11) exhibit higher mechanical strength, thermal stability, and chemical resistance^6–8^. While their mechanical performance has been studied extensively, considerably less information is available regarding their long-term durability, particularly when combined with natural fibers^9–11^. Compared to a conventional polyamide, such as PA6, PA5.10 has a higher relative methylene content per amide linkage in its polymer backbone, which reduces the overall hydrophilicity of the material^10,12^. As a result, PA5.10 exhibits lower water uptake and improved resistance to hydrolytic degradation under humid conditions, contributing to its enhanced long-term stability.

Moisture uptake in the hygroscopic polyamides leads to plasticization, reduced tensile strength, and weakened fiber-matrix adhesion^11–13^. Furthermore, hydrolytic chain splitting processes occur as absorbed water promotes cleavage of amide bonds, resulting in molecular weight reduction and deterioration of mechanical performance^13,14^. In addition to hydrolysis, thermo-oxidative and photo-oxidative degradation critically influence the durability of bio-based polyamides. Elevated temperatures increase molecular mobility and oxygen diffusion, thereby accelerating oxidative reactions and radical chain processes^15–17^. Likewise, exposure to UV radiation provides sufficient energy to break chemical bonds in the polymer backbone. In the presence of oxygen and moisture, photo-oxidation leads to chain splitting processes, embrittlement, and discoloration, typically involving Norrish type I and II reactions, which can be slowed down by the use of compatible stabilizer systems, like hindered amine light stabilizers (HALS)^18–24^. The incorporation of RCF further modifies the degradation behavior. Due to their hygroscopic nature, RCF act as local moisture reservoirs, which can accelerate hydrolysis at the fiber-matrix interface and impair adhesion^10,11,25^. Moreover, cellulose can undergo oxidative degradation under thermal and UV exposure, leading to a loss in single-fiber tensile strength and discoloration. Under elevated temperatures and prolonged UV exposure, the cellulose chains in RCF may exhibit depolymerization and oxidation, resulting in microfibril damage and fiber embrittlement. This leads to a decrease in load-bearing capacity of the RCF. These mechanisms contribute directly to a reduction in composite’s strength and a shift from ductile fiber pull-out to brittle fiber fracture^26,27^.

Against this background, the present study investigates the durability of neat and RCF-reinforced bio-based PA5.10. This polyamide is of particular interest because of its reduced moisture uptake and excellent oxygen barrier properties compared to conventional PA6^28,29^. To assess its long-term stability, the materials were subjected to thermal and photo-oxidative aging, followed by mechanical, thermal, chemical, and optical characterization. Special emphasis is placed on moisture absorption, crystallinity, molecular structure, and fiber-matrix interactions. Furthermore, different stabilization approaches are explored, including UV absorbers, HALS, and antioxidants, with the aim of identifying effective protection strategies against degradation. The novelty of this work lies in the systematic investigation of the PA5.10 composites reinforced with RCF and the assessment of how the fibers influence the aging resistance and property retention of the bio-based composite. In contrast to neat polyamides, the incorporation of cellulose-based fibers introduces additional interfaces and potential degradation pathways, which may significantly affect stabilization efficiency under combined UV and thermal stress. By combining multiple analytical techniques, the study therefore provides new insights into the interaction between stabilization strategies, fiber-matrix interfaces and degradation mechanisms in sustainable polyamide composites.

## Materials

This study examined six material batches, all based on AKROMID® NEXT 5.10 3 EXP nature (PA5.10), a 100% bio-based polyamide supplied by AKRO-PLASTIC GmbH (Niederzissen, Germany). It features a density of 1.08 g/cm³ and a melting temperature of 217 °C^30–32^. Regenerated cellulose fibers (RCF) from Cordenka GmbH & Co. KG (Obernburg am Main, Germany) were used as fiber-reinforcement. These bio-based and biodegradable fibers, produced with the viscose process, were incorporated as short fibers (2.5 mm in length) and feature a density of 1.5 g/cm³, an average fiber diameter of 12.65 μm, and a single fiber tensile strength of 830 MPa^33,34^.

In addition, two UV stabilizers were supplemented into the PA5.10 compounds, each with 1.0 wt%, to enhance resistance against thermo- and photo-oxidative degradation. Analyzing the effects of the different stabilizers offers insights into the key degradation mechanisms of the bio-based polyamide. AddWorks® IBC760 (IBC) from Clariant SE (Suelzbach, Germany) is a multifunctional stabilizer blend containing a benzotriazole-type UV absorber, hindered amine light stabilizers (HALS) based on bis(2,2,6,6-tetramethyl-4-piperidyl)sebacamide, and hindered phenolic antioxidants. The combination is designed to enhance both thermal and UV radiation stability and to reduce yellowing. Its chemical structure contains two sterically hindered piperidine moieties linked by a flexible aliphatic sebacamide, enabling long-term stabilization through a persistent radical-trapping mechanism. Upon exposure to UV radiation, photo-oxidative degradation of the polyamide generates alkyl and peroxyl radicals. IBC reacts with these radicals, forming stable nitroxyl radicals (R_2_NO*), which are capable of continuously deactivating newly formed radicals in the polymer matrix. This regenerative HALS cycle, supported by antioxidant activity, provides efficient long-term stabilization against UV- and thermo-oxidative degradation^22,35^. In contrast, LUBIO® UV 16 (LUBIO) from Schäfer Additivsysteme GmbH (Ludwigshafen am Rhein, Germany) is a hydroxyphenyl-triazine-based UV absorber. It is particularly suited for polyamide stabilization due to its strong absorption between 280 and 320 nm and its high compatibility. Its molecular structure incorporates a 2-(2-hydroxyphenyl)-1,3,5-triazine core, substituted with aryl and tert-butyl groups to enhance photostability. The aromatic system efficiently absorbs UV radiation and dissipates the energy as heat through non-radiative decay, thereby preventing molecular chain scission and inhibiting the initiation of photo-oxidative degradation^36,37^. In contrast to IBC, LUBIO acts predominantly as a passive UV screen with little chemical reactivity, which ensures long-term protection but does not interfere with radical propagation reactions. The additives used may contain processing aids that can affect rheology. However, this cannot be determined from the technical data sheets. Both neat PA5.10 and RCF-reinforced (20 wt%) compounds were stabilized and analyzed in this study.

## Sample preparation

The nomenclature used for the prepared test specimens defines PA5.10 as the neat bio-based polyamide AKROMID® NEXT 5.10 3 EXP nature and PA5.10RCF20 as PA5.10 reinforced with 20 wt% RCF. Samples containing stabilizers are indicated by the additive, with PA5.10_LUBIO and PA5.10_IBC containing 1 wt% LUBIO® UV 16 and 1 wt% AddWorks® IBC760 respectively. Combinations of fibers and stabilizers are written as PA5.10RCF20_LUBIO and PA5.10RCF20_IBC.

### Compounding process

PA5.10-based composites with 20 wt% RCF, partially including 1 wt% of IBC or LUBIO, were produced using a ZSE 18 HPe co-rotating twin-screw extruder (Leistritz Extrusionstechnik GmbH, Nuremberg, Germany) at 200 min^−1^ with an 18 mm screw diameter and 40D length. A fiber content of 20 wt% RCF was selected, corresponding to a volume fraction of approximately 12.5 vol%. This volume fraction is comparable to that of 30 wt% GF, a reinforcement commonly used in the automotive industry^3^. This choice enables meaningful comparison with existing reference materials. The screw configuration included kneading and conveying elements with increased free volume in front of the sidefeeder, positioned at 20D, followed by conveying elements to ensure minimal shear force on the RCF. Prior to compounding, PA5.10 was dried to < 0.1% moisture using a dry air system (TORO-Systems Dry Jet Easy dry air dryer, Gfk Thomas Jakob und Robert Krämer GbR, Igensdorf, Germany) for four hours at 80 °C, whereas the RCFs were dried at 105 °C for 24 h in a convection oven^38^. Material dosing was controlled via gravimetric feeders (Brabender Technology GmbH & Co. KG, Duisburg, Germany). The UV stabilizers were added to the matrix material by a separate feeder at the beginning of the compounding process. Processing temperatures ranged from 245 °C in the feeding zone to 220 °C at the nozzle, with metered melt temperatures at the nozzle of 240 °C and melt pressures of 40–80 bar. Minimum processing temperatures were chosen to protect the RCFs from thermal degradation^34,39^. The resulting strands were cooled with compressed air and pelletized into 3–4 mm granules using a Scheer SGS 25E pelletizer (Maag Germany GmbH, Grossostheim, Germany).

### Injection molding

In this study, type 1 A test specimens were produced in accordance with DIN EN ISO 527-2 using an Allrounder 320 C injection molding machine from Arburg GmbH & Co. KG (Lossburg, Germany), equipped with a 25 mm screw. The six batches were pre-dried at 80 °C for four hours to reduce moisture content below 0.1%, minimizing the residual moisture in the granules. Type 1 A test specimens were manufactured for both neat and RCF-reinforced batches. Injection molding was performed using a cold runner system, with a screw rotational speed of 15 m/min. The process parameters, including temperature profiles, are provided in Table 1.

**Table 1:** Parameters of the injection molding process.

Batch	Cylinder zone temperatures in °C						Injection pressure in bar	Packing pressure in bar	Cycle time in s	Cooling time in s
	1	2	3	4	5	Nozzle				
PA5.10	230	230	230	240	250	260	400	650	70	35
PA5.10RCF20	220	220	230	230	230	240	800	650	70	35
PA5.10_LUBIO	230	230	230	240	250	260	300	650	65	40
PA5.10RCF20_LUBIO	220	220	230	230	230	240	1000	650	65	40
PA5.10_IBC	230	230	230	240	250	260	300	650	65	40
PA5.10RCF20_IBC	220	220	230	230	230	240	1000	650	65	40

### Thermo- and photo-oxidative aging conditions

To investigate the effects of thermal and UV-induced aging, as well as the influence of the UV stabilizers, a total of nine aging conditions were applied. These included a non-aged reference and thermal storage at 23 °C, 50 °C, 70 °C, and 90 °C, each conducted with and without UV radiation. The non-aged specimens were sealed immediately after injection molding in aluminum composite bags to prevent exposure to ambient humidity. Storages were performed in climate chamber type 3433/18 (Feutron Klimasimulation GmbH, Langenwetzendorf, Germany) equipped with a SOL500 radiation module. The SOL module simulates natural sunlight with an irradiance of 1000 ± 10% W/m^2^ over the spectral range of 305 to 2800 nm, measured at a distance of 1.0 m from the light source. The light source used is a 500 W metal halide spotlight. The radiation source consists of a 500 W metal halide spotlight, providing a solar-like spectrum relevant for polymer photodegradation. UV irradiation was applied to induce photo-oxidative degradation processes, while the combination with elevated temperatures (23, 50, 70, and 90 °C) allowed the acceleration of thermo-oxidative and photo-oxidative aging mechanisms under controlled laboratory conditions.

Each storage condition, was applied for a duration of 168 h. Subsequently, all specimens were conditioned under standard climate according to ISO 291 (23 °C, 50%rH) for an additional 168 h to ensure equilibration before testing.

## Methods

All tests were conducted at standard climate (23 °C,50%rH) according to ISO 291^40^. The methods applied are described in detail in the following subsections.

### Gravimetric analysis of the moisture uptake

The moisture uptake *c* has been analyzed by measuring the weight of 15 type 1 A test specimens each before (*wt*_*1*_) and after storage (*wt*_*2*_), using Eq. (1). The electronic precision scale CUBIS II by Sartorius AG (Goettingen, Germany) with 10^− 3^ g accuracy was used in this study.

1 $$\:c=\:\frac{{wt}_{2}\:-\:{wt}_{1}}{{wt}_{1}}*100\:\%$$

### Thermal and rheological analysis

To determine the crystallinity of the specimens, Differential Scanning Calorimetry (DSC) was carried out using the DSC Q1000 module from TA Tools (New Castle, USA). Samples were heated at a rate of 20 K/min up to 300 °C under inert nitrogen atmosphere. The first heating cycle was evaluated, as it reveals the influence of degradation through changes in crystallinity and microstructure, visible in the first melting peak^41^. The area under the melting peak corresponds to the melt enthalpy (*ΔH*), which was used to calculate the degree of crystallinity *X*_*c*_ for the non-reinforced samples according to Eq. (2)^41^. A value of 208 J/g for the melt enthalpy of 100% crystalline polyamide (*ΔH*_*m*_) was taken from literature^42^. DSC measurements were limited to the non-reinforced PA5.10, as the presence of RCFs can distort the results. Samples were extracted from the cross-section of the parallel section of the type 1 A test specimen to account for the influence of the amorphous surface layer.

2 $$X_{c} = \frac{{\Delta H}}{{\Delta H_{m} }}*100\%$$

The melt volume rate (MVR) was determined using a Meltfixer 2000 (Thermo Haake, Karlsruhe, Germany) in accordance with ISO 1133-1. Tests were conducted at 250 °C under a 2.16 kg load, using samples of the parallel section of two type 1 A test specimens per batch and condition. MVR testing was performed only on neat PA5.10, as fiber-reinforcement can affect melt viscosity and compromise result comparability. Only non-aged as well as 23 °C and 70 °C aged specimens (with and without UV exposure) were analyzed as this is where the greatest differences in the results of the tensile tests were visible.

### Fourier transform infrared spectroscopy

Fourier Transform Infrared Spectroscopy (FTIR) analyses were performed on three specimens per batch using a Shimadzu IRAffinity-1 S spectrometer (Kyōto, Japan) equipped with a germanium crystal. Three specimens per batch were examined within a spectral range of 3400–1000 cm^−1^ using the attenuated total reflection (ATR) mode with 32 scans accumulated per spectrum at a resolution of 4 cm^−1^. In this study, FTIR measurements were limited to the parallel section of the non-reinforced PA5.10 (neat and stabilized) type 1 A test specimens, as the presence of RCFs was found to interfere with infrared signal intensity and data quality.

### Optical and surface analysis

The surface energy was determined using the sessile drop method with droplet volumes of 1–2 µL, utilizing distilled water and diiodo-methane as test liquids. Measurements were conducted with a Drop Shape Analyzer DSA 20B (Krüss GmbH, Hamburg, Germany). For each non-reinforced batch and condition, ten droplets were applied and analyzed across two specimens, using the parallel section of the type 1 A test specimen. The surface energy components, including the polar and dispersive share, were calculated using the Owens-Wendt-Rabel-Kaelble (OWRK) method based on the measured contact angles. The fiber-reinforced batches were not measured, as they present a rougher, more heterogeneous surface texture, which would introduce variability and make the measurements less reproducible.

Color properties were evaluated in accordance with the guidelines of the Commission Internationale de l’Eclairage (CIE) using the CIELAB color space, which defines the three coordinates L* (lightness), a* (red-green axis), and b* (yellow-blue axis). It was measured with an Ultra Scan Pro spectrophotometer (HunterLab, Murnau, Germany). Ten specimens per non-reinforced batch and condition were tested, with measurements taken from the parallel section of the type 1 A test specimens. As the primary focus lies on the yellowing of the specimens caused by storage at elevated temperatures and exposure to UV radiation, the analysis is limited to changes in the b*-value. The fiber-reinforced batches were not measured, as the change in yellowness is not clearly visible in the brownish color.

The fracture surface morphology of selected RCF-reinforced specimens, non-aged and aged at 70 °C under UV exposure, was analyzed using scanning electron microscopy (SEM), subsequent to the tensile tests. Images were captured using an MV2300 microscope (CamScan Electron Optics Services, Ottawa, Canada) at 10 kV accelerating voltage and 500x magnification. Prior to imaging, fracture surfaces of the type 1 A test specimens were sputter-coated with gold to ensure conductivity and image quality.

### Mechanical characterization

Type 1 A tensile specimens, prepared in accordance with DIN EN ISO 527, were tested using a UPM 1446 universal testing machine from ZwickRoell GmbH & Co. KG (Ulm, Germany) equipped with a 10 kN load cell and adjustable extensometers during the measurement of the young’s modulus. The non-reinforced specimens were tested at a higher crosshead speed of 25 mm/min, while the fiber-reinforced batches were tested at 5 mm/min to account for the significant differences in their mechanical behavior. RCF-reinforced composites exhibit higher stiffness and strength at lower ductility compared to the neat polyamide. Reducing the strain rate for the RCF-reinforced type 1 A test specimen minimizes dynamic effects and allows for a more controlled measurement of their brittle fracture behavior. For each batch and condition, five specimens were analyzed to determine tensile strength and elongation at break. Due to the very high ductility of the non-reinforced PA5.10 specimens, no specimen failure occurred within the available machine travel and extensometer range. To ensure reproducible and comparable stress–strain data across all aging conditions, tensile testing of the non-reinforced specimens was therefore limited to an elongation of 80%. The resulting data were used to evaluate degradation-related changes in mechanical behavior rather than absolute failure properties.

Instrumented notched impact tests (notch type A, DIN EN ISO 179-2) were carried out on ten specimens per RCF-reinforced batch and condition. Notching was performed using the NOTCHVIS notching machine from CEAST/INSTRON (Darmstadt, Germany). The Charpy impact strength, including impact work, fracture work, and residual work, was measured using a Charpy impact tester from Zwick GmbH & Co. KG (Ulm, Germany) equipped with a 5 J pendulum.

### Linear polynomial regression modeling and R^2^ evaluation

To investigate the correlation between crystallinity and moisture absorption on the tensile strength, elongation at break, and melt volume rate, three-dimensional polynomial regression modeling was applied using *Python 3.11* (Python Software Foundation, Wilmington, USA). Each target variable was modeled as a function of crystallinity and moisture absorption using third-degree polynomial regression. A cubic model was deliberately chosen as a compromise between capturing nonlinear relationships and avoiding overfitting, which can occur with higher-order polynomials that adapt too strongly to random noise in the data. This approach ensures that the regression highlights the general trends in property evolution while maintaining physical interpretability. Predictor variables were expanded using *PolynomialFeatures* from *scikit-learn*^43^ to include interaction and non-linear terms. A linear regression model was fitted to the transformed dataset. Model performance was assessed using the coefficient of determination R^2^. All curve fittings and visualizations were performed using *NumPy*^44^, *Matplotlib*^45^, and *pandas*^46^. ChatGPT (OpenAI, San Francisco, USA) was used as a supportive tool for the *Python* scripts in this study, assisting with code structuring and performance optimization. The script was carefully evaluated, tested, and adapted to the specific needs of the analysis. A 3rd-degree polynomial regression equation with crystallinity (*X*_*c*_) and moisture absorption (*M*) as the latent factors can be expressed with the following Eq. (3)^47,48^.

3 $$y_{i} = a_{i} + b_{{1i}} X_{c} + b_{{2i}} M + c_{{1i}} X_{c}^{2} + c_{{2i}} X_{c} M + c_{{3i}} M^{2} + d_{{1i}} X_{c}^{3} + d_{{2i}} X_{c}^{2} M + d_{{3i}} X_{c} M^{2} + d_{{4i}} M^{3}$$

This equation accounts for the linear, quadratic and cubic effects on the target variables tensile strength, elongation at break, and melt volume rate.

## Results and discussion

### Moisture absorption

**Fig. 1:**
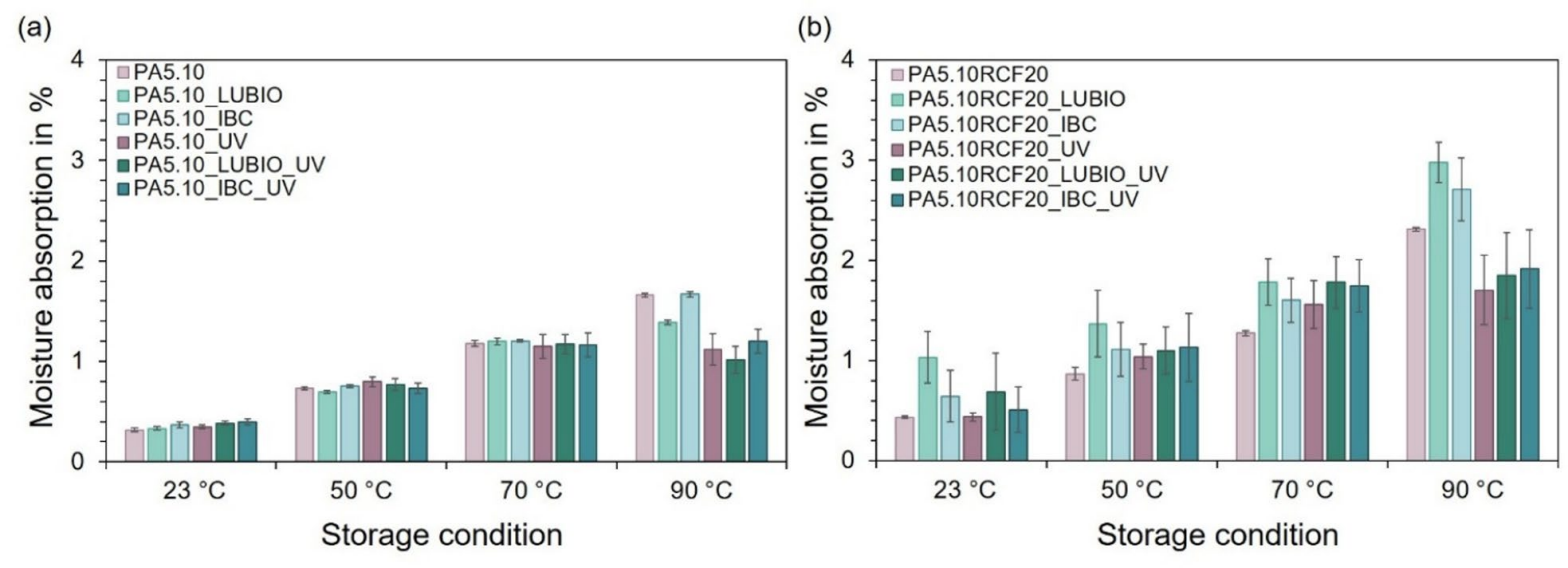
The column charts illustrate the moisture absorption behavior of the type 1 A test specimens after exposure to the defined storage conditions. In (**a**), the moisture absorption in % of the three non-reinforced batches is displayed in relation to storage temperatures of 23 °C, 50 °C, 70 °C, and 90 °C, each at 50%rH for 168 h, with and without UV exposure. In (**b**), the same experimental conditions are applied to the three RCF-reinforced batches.

At the beginning, the moisture absorption of the six batches due to the thermo- and photo-oxidative storage will be discussed. Figure 1 (a) shows an increase in moisture absorption with elevated storage temperature across all non-reinforced batches. A clear trend of increasing moisture uptake can be observed with rising storage temperature (23, 50, 70, and 90 °C) under 50% relative humidity, both with and without UV irradiation and independently of the presence of RCF, can be attributed to accelerated diffusion processes at elevated temperatures. Higher temperatures enhance molecular mobility within the polyamide matrix and the fibers, resulting in faster moisture penetration and a higher equilibrium moisture uptake^10,25^. However, the UV exposed specimen display a lower moisture absorption, which is particularly noticeable with the specimens stored at 90 °C. The largest increase of the thermal stored specimen can be observed for PA5.10 and PA5.10_IBC, which amounts to 1.7%. In comparison, the maximum moisture absorption for the UV exposed specimens only corresponds to 1.2%. In comparison with other storage conditions, e.g. in a water bath or in acidic chemicals, this is a quite low value for PA5.10^49^. This is due to UV-induced photo-oxidative degradation on the polyamide’s surface, which leads to chemical changes such as molecular chain splitting processes due to oxidation as well as cross-linking. These changes reduce the number of hydroscopic sites and form a less permeable surface layer that decreases water diffusion. Thus, both a reduction of hydrophilic surface groups and a slower moisture diffusion contribute to the lower water uptake under UV exposure. As a result, the PA5.10 absorbs less moisture under UV exposure compared to the same conditions without UV^50^. It should be noted, however, that UV-induced surface embrittlement or the formation of microcracks could locally counteract this effect, potentially allowing moisture to penetrate at damaged sites. In addition, at 90 °C, the LUBIO stabilized batches absorb significantly less moisture than the neat or IBC-stabilized PA5.10. This is due to stabilizer-induced changes (see Chap. 2), resulting in a smoother, more hydrophobic surface^21^.

In Fig. 1 (b) the difference between UV and no UV exposure is even more noticeable, as the RCF absorb significantly less moisture due to the surface layer. Generally, the overall moisture absorption is noticeably higher with the RCF-reinforced batches, which can be attributed to the hygroscopic material behavior of the RCF as well as by the capillary effect of natural fibers^9,10,51^.

### Thermal analysis

**Fig. 2:**
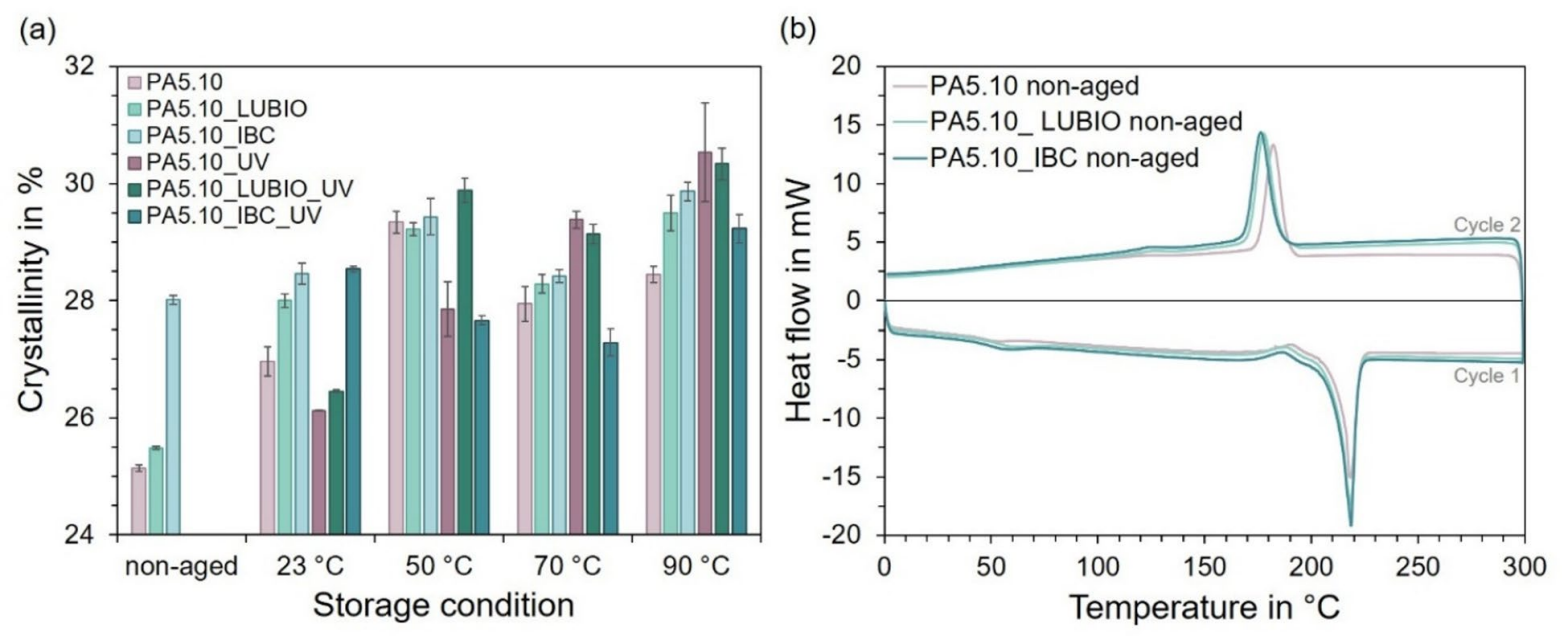
Differential Scanning Calorimetry results of the non-reinforced batches under the thermal aging conditions. In (**a**), the degree of crystallinity in % is displayed. In (**b**), heat flow in mW is shown as a function of temperature for the non-aged reference samples.

The DSC analysis of the non-reinforced batches subsequent to the thermo- and photo-oxidative aging reveals distinct trends in both crystallinity and thermal transitions as well as synergistic effects. The corresponding crystallinity data for the six non-reinforced PA5.10 batches are listed in Table 2 and are discussed in the following section. An overall increase in crystallinity is observed, attributed both to the use of additives and the influence of the elevated storage temperatures due to annealing processes and degradation. At 50 °C, near T_g_, a pronounced rise in crystallinity suggests temperature-induced reordering effects^51^. The increased crystallinity, which can be seen in the non-aged PA5.10 samples upon addition of LUBIO and IBC, can be attributed to stabilizer-induced changes in the crystallization kinetics during processing. Low-molecular-weight stabilizers act as nucleating agents, thereby promoting more efficient crystal formation during cooling^22,35–37^. As shown in Fig. 2 (a), the degree of crystallinity further increases across the samples, except for the PA5.10_IBC specimens under UV exposure. Generally, in non-aged condition, the stabilizer incorporation results in a high increase in crystallinity. This rise, visible after the storage at elevated temperatures, is attributed to the combined effects of elevated storage temperatures on one hand, which promote annealing and reorganization within the polymer matrix at temperatures above T_g_^10,52^. On the other hand, chain splitting processes result in shorter molecular chains which crystallize better at temperatures above T_g_^25^. Notably, at 50 °C, close to T_g_, a significant increase in crystallinity is observed, indicating temperature-driven structural reorganization^52^. Furthermore, the UV exposure appears to contribute to this effect by inducing chain splitting processes, which facilitates better packing and alignment of polymer segments into crystalline domains^53^.

Figure 2 (b) presents the corresponding heat flow as a function of temperature for the non-aged reference materials. Here, a noticeable shift of T_g_ from 54.54 °C (non-aged) to 62.52 °C (LUBIO) and 57.79 °C (IBC) can be seen. In addition, a decrease in crystallization temperature is observed, suggesting modified crystallization kinetics due to the stabilizers. The enhanced crystallinity observed in Fig. 2 (a) is also reflected in the melting behavior during the first heating cycle, where a more pronounced melting peak is evident. *Shacklefold et al.* also observed an increase in melt enthalpy, which implies that significant changes in crystal structure within the polyamide’s surfaces had taken place during the UV exposure. The observed change of the crystalline phase is in line with chemical alterations caused by UV exposure according to the literature, as it is known that UV radiation influences the distribution of hydrogen bonds within the polymer matrix affecting the degree of crystallinity^54^.

**Table 2:** Degree of crystallinity of the six non-reinforced PA5.10 batches under the investigated conditions.

Storage Condition		PA5.10	PA5.10_UV	PA5.10_ LUBIO	PA5.10_ LUBIO_UV			PA5.10_ IBC	PA5.10_ IBC_UV
non-aged	mean	25,139	25,139	25,483	25,483			28,010	28,010
	SD	0,053	0,053	0,031	0,031			0,077	0,077
23 °C	mean	26,966	26,127	27,998	26,459			28,457	28,536
	SD	0,361	0,012	0,147	0,017			0,269	0,050
50 °C	mean	29,339	27,856	29,216	29,887			29,430	27,659
	SD	0,113	0,673	0,846	0,209			0,618	0,077
70 °C	mean	27,942	29,380	28,281	29,135			28,418	27,288
	SD	0,139	0,659	0,161	0,750			0,106	0,226
90 °C	mean	28,447	30,531	29,498	30,337			29,865	29,228
	SD	0,139	0,839	0,300	0,769			0,159	0,238

### Molecular structure

**Fig. 3:**
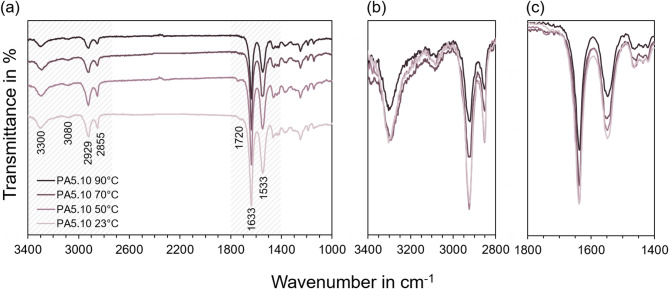
In (**a**) the ATR-FTIR spectra of PA5.10 after storage at 23 °C, 50 °C, 70 °C, and 90 °C can be seen. The graph shows transmittance in % on the y-axis as a function of wave length in cm^−1^. In (**b**) and (**c**) detailed views of the spectra between 3400–2800 cm^−1^ and 1800–1400 cm^−1^ can be observed with superimposed signals.

The ATR-FTIR results provide insights into molecular changes occurring in PA5.10 during storage under thermo- and photo-oxidative conditions. Variations in the spectra enable a qualitative assessment of structural modifications such as changes in crystallinity, molecular chain splitting, and (thermo-) oxidation. The spectra are presented in a stacked representation to allow comparison of the multiple aging conditions. They are evaluated in the following based on the relative intensity variations and the appearance or modification of polyamide-specific bands. Figure 3 (a) shows the transmittance spectra between 3400 cm^−1^ and 1000 cm^−1^ for specimens stored at 23 °C, 50 °C, 70 °C, and 90 °C. The sample stored at 23 °C serves as a reference prior to thermal aging. With increasing storage temperature, a general change in spectral intensity is observed, most pronounced at 2929 cm^−1^ and 2855 cm^−1^, corresponding to C–H stretching vibrations. This can be seen in more detail in Fig. 3 (b), as the superimposed signals show a clear trend in increasing intensity. The bands belong to the CH_2_ stretching region, where partially overlapping peaks can be associated with methylene groups in amorphous and more ordered chain conformations of the polyamide structure. The variations may reflect a change in the amorphous-to-crystalline ratio of the PA5.10^28^. In particular, a relative shift in intensity between these contributions may indicate structural rearrangements and increased chain packing during thermal exposure. However, it should be noted that in ATR mode, apparent intensity changes can also result from altered surface contact with the ATR GE-crystal as crystallinity increases. A more detailed view of the region between 1800 and 1400 cm^−1^ in Fig. 3 (c) reveals modifications in bands associated with C = O and N–H vibrations. A weak shoulder around 1720 cm^−1^ is indicative of carbonyl formation, which is typically linked to oxidation of polyamides^55–57^. Such carbonyl-containing species may arise from thermo-oxidative chain splitting processes leading to the formation of ketones, aldehydes, or carboxylic groups. The presence of this shoulder therefore serves as an indicator of oxidation-related chemical modifications during aging. The apparent reduction of this shoulder at higher temperatures may result from overlapping effects of structural reorganization and changes in band intensity within the surrounding amide region, which can partially mask the signal^56^. Slight changes near 1533 cm^−1^ may point to hydrolytic effects or variations in hydrogen bonding. This band corresponds to the amide II vibration of the polyamide backbone and is known to be sensitive to changes in hydrogen bonding interactions within the polymer structure. Variations in its intensity may therefore reflect modifications in intermolecular hydrogen bonding or minor hydrolysis-related changes occurring during aging^52^. Overall, these observations suggest thermally induced structural reorganization as well as hints of hydrolytic degradation.

**Fig. 4:**
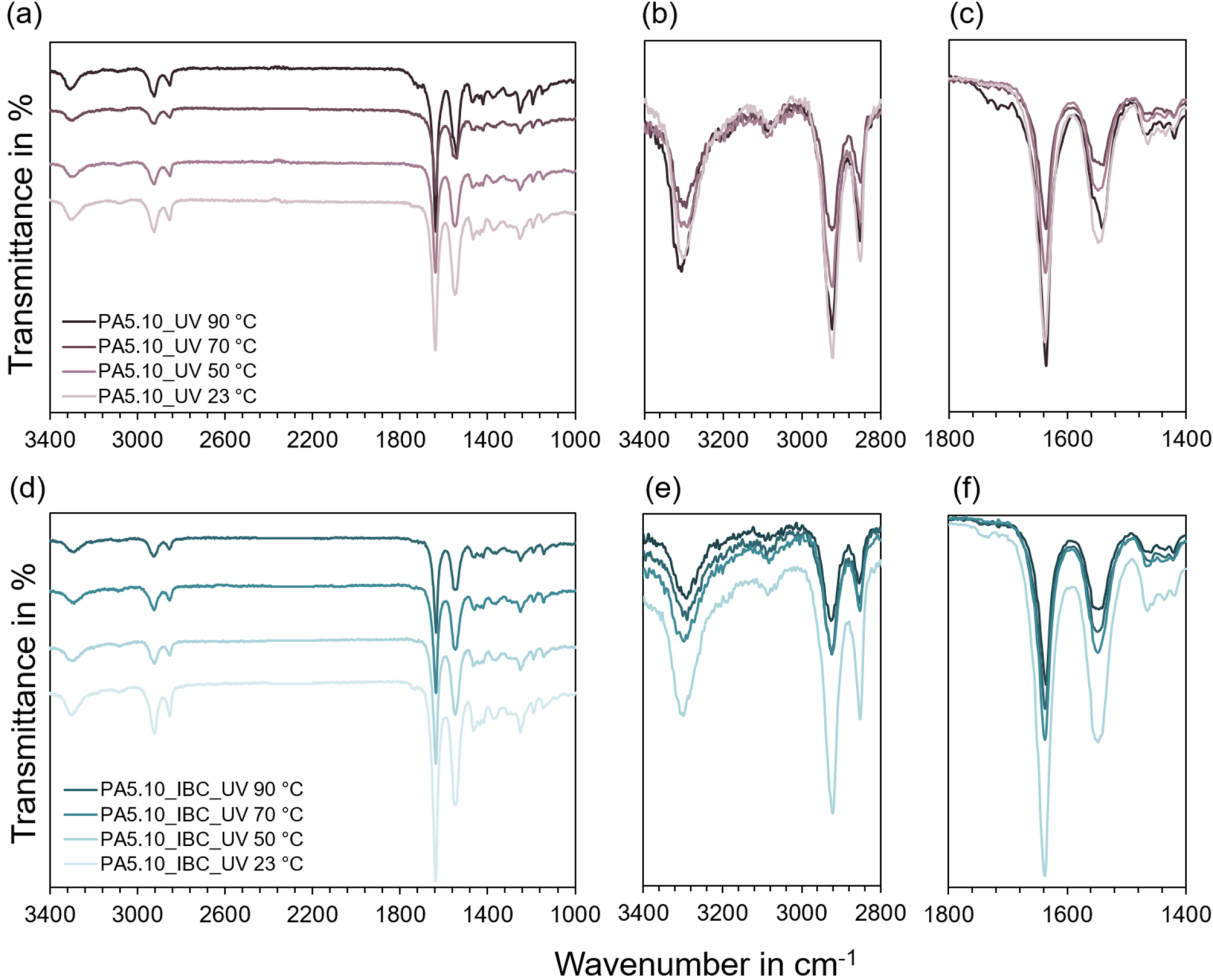
In (**a**), the ATR-FTIR spectra showing transmittance in % as a function of the wave length in cm^−1^ for UV-stored PA5.10 specimens at 23 °C, 50 °C, 70 °C, and 90 °C can be seen. (**d**) displays the corresponding spectra of UV-stored PA5.10_IBC specimens. In (**b**), (**c**) as well as (**e**), (**f**) detailed views of the spectra between 3400–2800 cm^−1^ and 1800–1400 cm^−1^ can be observed with superimposed signals.

In Fig. 4 (a), ATR-FTIR spectra of UV-exposed PA5.10 specimens stored at 23 °C, 50 °C, 70 °C, and 90 °C are presented. Up to 70 °C, the spectra resemble those of Fig. 3 (a), with moderate intensity variations at 3300, 2929, 2855, 1633, and 1533 cm^−1^. At 50 °C and 70 °C, a general decrease in spectral intensity is observed across all bands. Unexpectedly, at 90 °C a decrease in intensity can be seen, especially in the detailed views in Fig. 4 (b) and (c). This can be explained, as ATR spectra are strongly affected by surface morphology and refractive index, which influence the overall transmission. Therefore, the reduction cannot be solely attributed to cross-linking reactions as previously proposed by *Hurtado et al.* and *Marek and Verney*^21,58^. Nevertheless, the appearance of a shoulder near 1720 cm^−1^ for the 90 °C sample suggests the onset of oxidation, consistent with typical carbonyl formation in photo-oxidation^21^. The combined UV and thermal exposure accelerate such oxidation reactions due to synergistic effects, as photo-oxidation of polymers is thermally activated with activation energies of approximately 60–70 kJ/mol.

Figure 4 (d) shows the ATR-FTIR spectra of UV-exposed PA5.10_IBC specimens. At 23 °C, slightly lower overall transmittance is observed compared to the neat PA5.10, which can be attributed to the presence of UV absorbers within the IBC formulation (see Chap. 2) rather than to differences in molecular chain length. With increasing temperature, spectral changes, which can be seen in detail in Fig. 4 (e) and (f), follow a similar trend to those in Fig. 3 (b) and (c). The absence of the pronounced spectral decrease seen in Fig. 4 (c) at 90 °C indicates that the stabilizer system effectively mitigates the surface modifications observed in the non-stabilized PA5.10, supporting the beneficial interaction of HALS, UV absorbers, and antioxidants in suppressing thermo-photo-oxidative effects.

### Mechanical properties

**Fig. 5:**
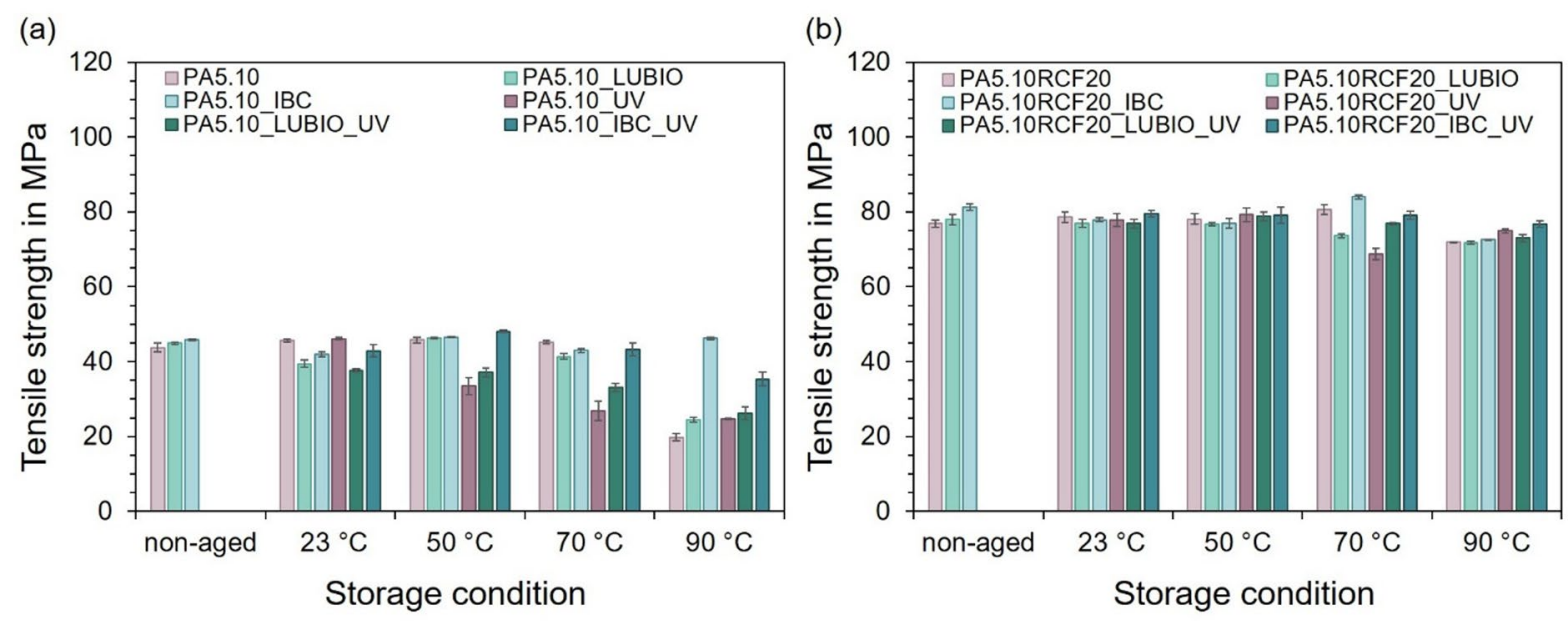
The column charts show the tensile strength of the type 1 A test specimens after exposure to the defined storage conditions. In (**a**), the tensile strength in MPa of the three non-reinforced batches is displayed in relation to the non-aged reference and after the storage. In (**b**), the same experimental conditions are applied to the three RCF-reinforced batches.

Following the characterization of the combined effects of elevated temperatures and UV radiation using moisture absorption, crystallinity and molecular structure, the impact on the mechanical properties is discussed below, starting with the results of the tensile test. Figure 5 presents the tensile strength results of type 1 A test specimens following exposure to the thermo- and photo-oxidative storage conditions. In Fig. 5 (a), the tensile strength in MPa of the three non-reinforced PA5.10 batches are compared over the range of the storage temperatures 23 °C, 50 °C, 70 °C and 90 °C, each at 50%rH for 168 h, followed by one week of conditioning in standard climate. The non-aged specimens serve as a reference. For the neat PA5.10 specimens, only minor variations in tensile strength are observed between the non-aged state and storage temperatures up to 70 °C, indicating that the polymer matrix remains largely stable under these conditions. A more pronounced reduction in tensile strength occurs after storage at 90 °C, suggesting that significant thermo-oxidative degradation mainly takes place at the highest storage temperature^13^. Up to 70 °C, the degradation of all UV exposed specimens is stronger in comparison to the temperature stored specimens. However, the neat PA5.10 specimens display a strongly decreased tensile strength subsequent to the storage at 90 °C without UV exposure. It is possible that the higher tensile strength can be explained by the results of the FTIR measurement in Fig. 4 (a), as the possible cross-linking processes observed here may lead to a higher tensile strength^21^. A similar trend is observed for the PA5.10_LUBIO formulation, where only small fluctuations in tensile strength occur up to 70 °C, followed by a clearer reduction at 90 °C, indicating that significant degradation effects mainly develop at the highest storage temperature. In contrast, the PA5.10_IBC batch in Fig. 5 (a) exhibits remarkable thermal stability, with no significant reduction in tensile strength across the elevated temperatures. However, a slight decrease is evident after UV exposure at 90 °C, suggesting starting photo-oxidative degradation, which is attributable to surface-level oxidation or molecular chain splitting processes induced by the UV radiation as well as the thermal stress^21,22^.

Figure 5 (b) displays the corresponding results for the three RCF-reinforced batches, subjected to the same environmental conditions. While a similar temperature-dependent decrease is evident, the overall loss in tensile strength is noticeably lower in comparison to the non-reinforced PA5.10 in Fig. 5 (a). This is due to aging-induced embrittlement as well as the increased moisture absorption resulting in plasticization processes^54,59^. Furthermore, *Cundiff et al.* observed that a high moisture content accelerates thermo-oxidative degradation, decreasing the materials strength by further accelerating the auto-oxidation cycle^50^. Nevertheless, the overall reduction is considerably lower compared to the neat PA5.10 type 1 A test specimens shown in Fig. 5 (a). This enhanced stability is primarily attributed to the presence of natural antioxidants inherent in cellulose-based fibers, which are known to scavenge free radicals and thus mitigate polymer degradation during thermal and UV storage. Additionally, the reinforcing effect of the RCF may contribute to a mechanical stabilization of the composite structure, further reducing the loss in tensile strength under accelerated aging conditions^8,10,25^.

**Fig. 6:**
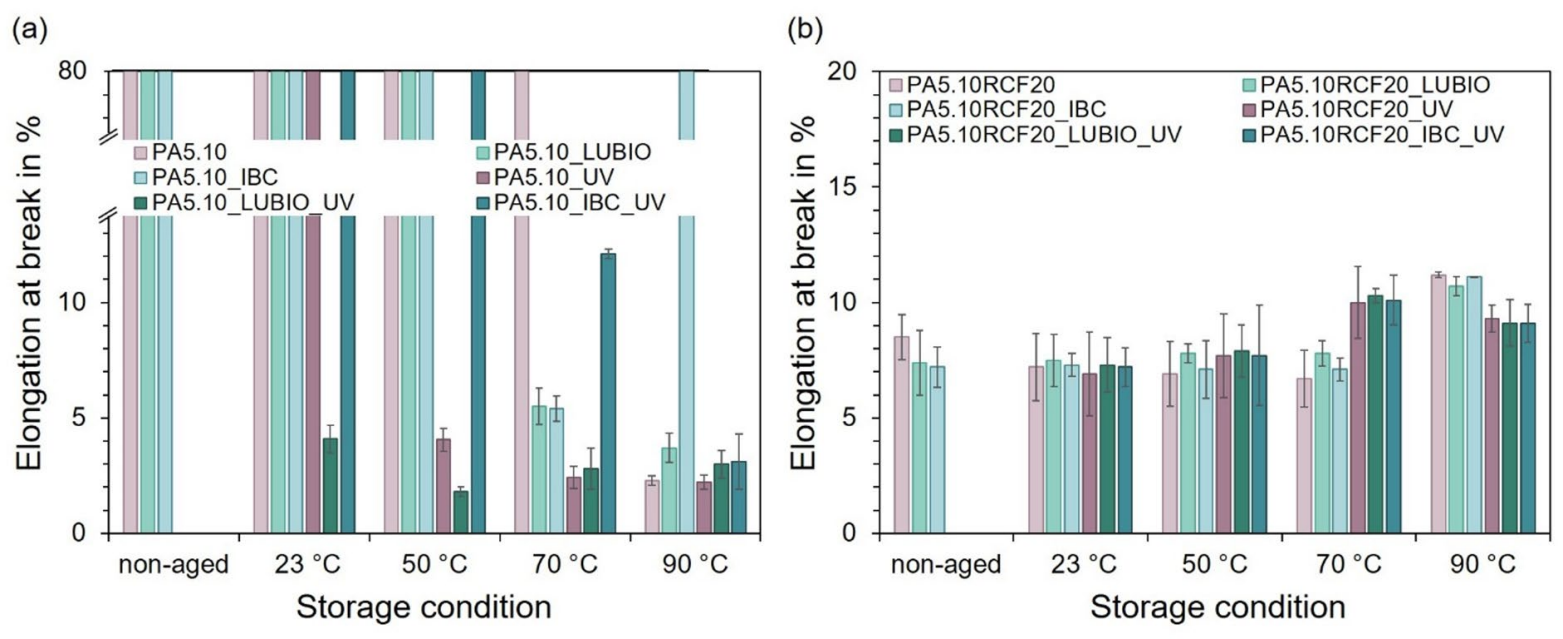
In (**a**) the elongation at break in % of the three non-reinforced PA5.10 batches, shown relative to the non-aged reference, after storage at 23 °C, 50 °C, 70 °C, and 90 °C at 50% relative humidity for 168 h, with and without UV-impact, can be seen. The elongation decreases with increasing storage temperature, due to embrittlement and is significantly affected by the UV exposure^13^. (**b**) shows the elongation at break of the three RCF-reinforced batches.

In addition, Fig. 6 shows the results of the elongation at break similarly to the results of the tensile strength in Fig. 5. It illustrates the elongation at break in % of the tested type 1 A specimens subsequent to the exposure to the thermo- and photo-oxidative storage conditions. In Fig. 6 (a), the three non-reinforced PA5.10 batches are compared in relation to the non-aged reference and after being stored at 23 °C, 50 °C, 70 °C, and 90 °C, each at 50% relative humidity for 168 h, both with and without UV exposure. A progressive decrease in elongation at break is observed with increasing storage temperature, which can be attributed to thermally-induced embrittlement of the PA5.10 matrix^13^. This trend is significantly intensified by the influence of UV radiation, due to photo-oxidative degradation processes such as molecular chain splitting and cross-linking, which reduce the ductility of the material significantly^24^.

The significantly reduced elongation at break observed for PA5.10_IBC after aging at 70 °C may be related to temporary embrittlement caused by thermo-oxidative degradation processes at intermediate temperatures. At higher temperatures (90 °C), increased molecular mobility and possible structural reorganization may partly restore ductile behavior, which results in higher elongation values. Among the non-reinforced batches, PA5.10_IBC exhibits the least reduction in elongation at break, suggesting enhanced resistance to thermo- and photo-oxidative degradation effects, due to the stabilizing effects of the HALS as well as the antioxidants (see Chap. 2) that improve the polyamide’s thermal and UV stability^22^.

In Fig. 6 (b), the elongation at break of the three RCF-reinforced PA5.10 batches can be seen. In contrast to the behavior observed in the non-reinforced type 1 A test specimens, the RCF-reinforced batches show an increase in elongation at break, following the thermo- and photo-oxidative aging. This counterintuitive trend is attributed to the elevated moisture uptake capacity of the RCF, which is visible in Fig. 1 (b). The absorbed moisture leads to increased molecular mobility, resulting in improved durability of the RCF and therefore a higher elongation under tensile stress^25^. This effect highlights the complex interplay between fiber-reinforcement, environmental exposure and resulting mechanical properties in NFC, which has also been observed in previous studies^10,11,25^. Subsequent to the storage at 90 °C under UV exposure, a reduced elongation at break of the RCF-reinforced batches can be seen. This may be due to the effect of embrittlement of the PA5.10 matrix, which starts to occur here, whereby the elongation at break decreases again, similar to the non-reinforced PA5.10 batches in Fig. 6 (a). Furthermore, it is noticeable that the stabilizers IBC and LUBIO have no positive nor negative effect on the elongation of the RCF-reinforced batches, as the results do not differ significantly from each other.

**Fig. 7:**
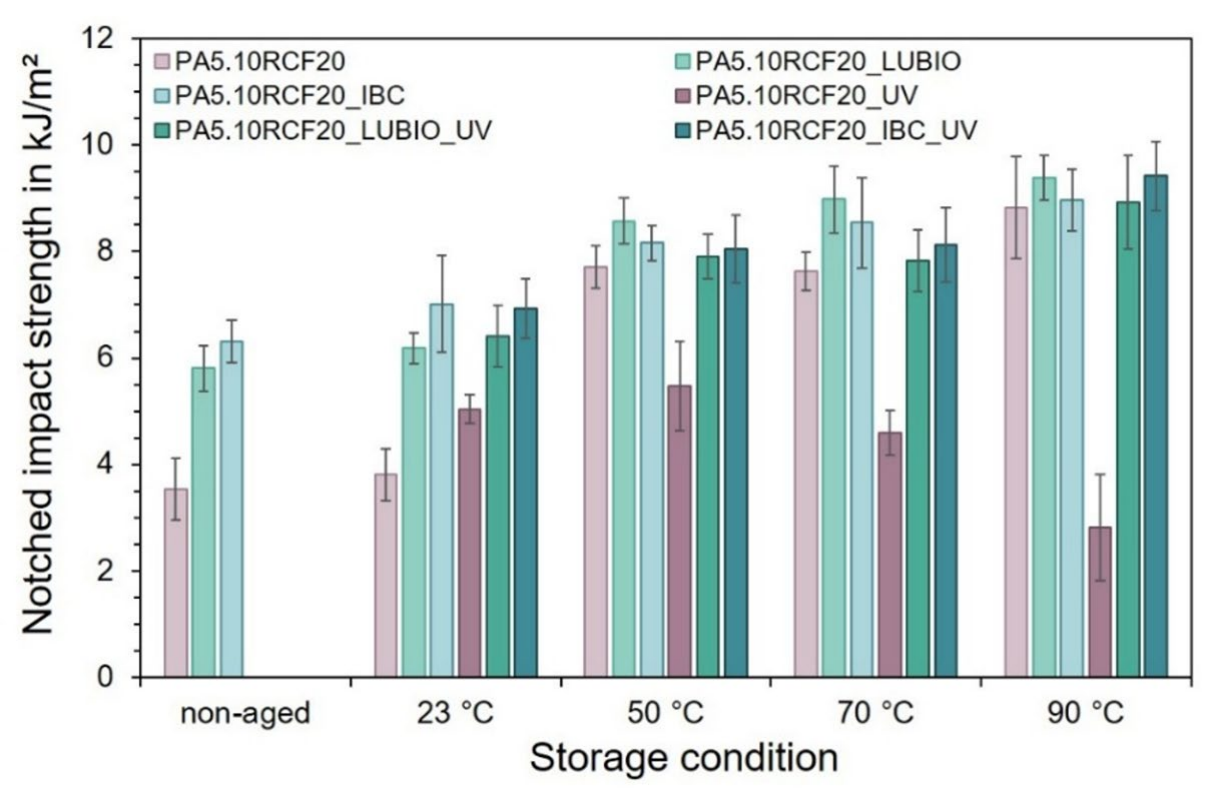
The notched impact strength in kJ/m^2^ of the three RCF-reinforced PA5.10 batches, is shown in relative to the non-aged reference, after storage at 23 °C, 50 °C, 70 °C, and 90 °C at 50% relative humidity for 168 h, with and without UV exposure.

In addition to the results of the tensile test, Fig. 7 shows the notched impact strength in kJ/m^2^ of the three RCF-reinforced PA5.10 batches. In general, an increase in notched impact strength is observed with rising storage temperature along all batches except for the UV exposed PA5.10RCF20. This behavior can be primarily attributed to the hygroscopic nature of the RCF, whereby they absorb moisture, which acts as a plasticizer, enhancing the ductility and energy dissipation capacity of the RCF-composite under impact load^60^. This is consistent with the increased moisture content measured for the RCF-reinforced batches, as shown in Fig. 1 (b) as well as the increased elongation at break in Fig. 6 (b). However, this does not apply to the UV exposed PA5.10RCF20 batch, as there is a decrease in notched impact strength from 70 °C. This reduction is likely due to the synergistic effects of the thermo- and photo-oxidative degradation, which compromises the structural integrity of the PA5.10 itself, which could also be seen in Figs. 4, 5 and 6 (a)^26,27^. UV exposure can also lead to microcracking and surface embrittlement, partially offsetting the plasticizing effect of the absorbed moisture^13,61^.

### Optical and surface analysis

**Fig. 8:**
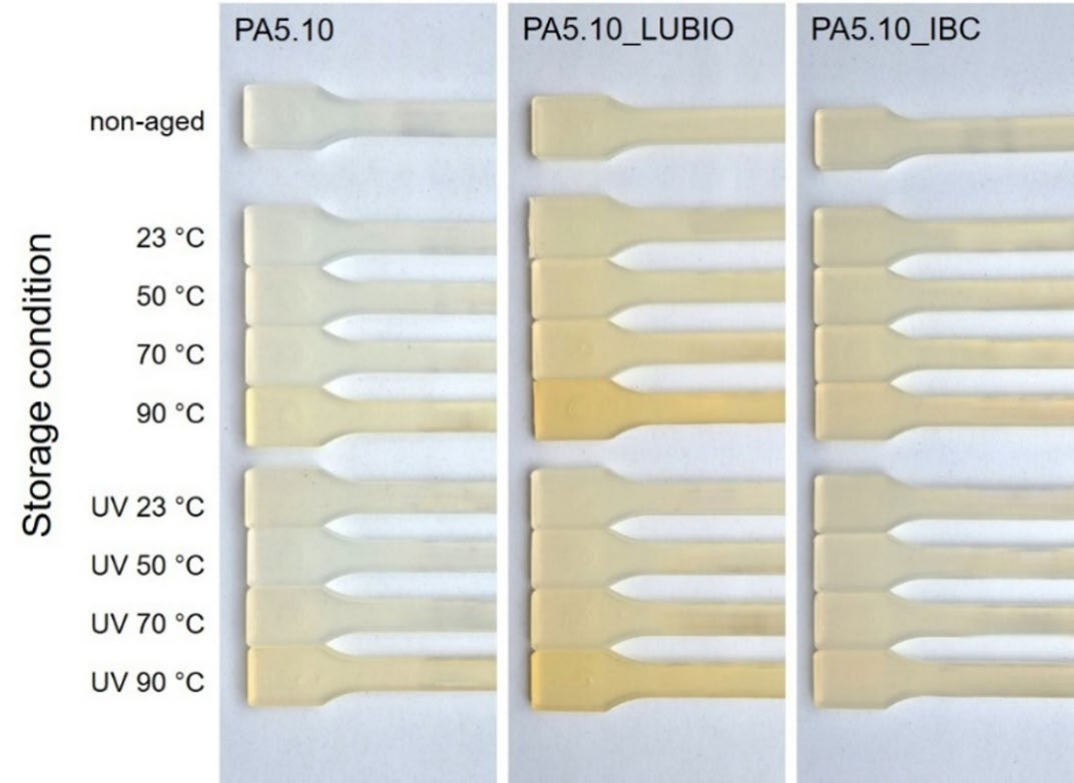
Pictures of the three non-reinforced PA5.10 batches before (non-aged) and after thermo- and photo-oxidative storage. PA5.10 shows noticeable yellowing, particularly with the 90 °C and UV 90 °C stored specimens.

**Fig. 9:**
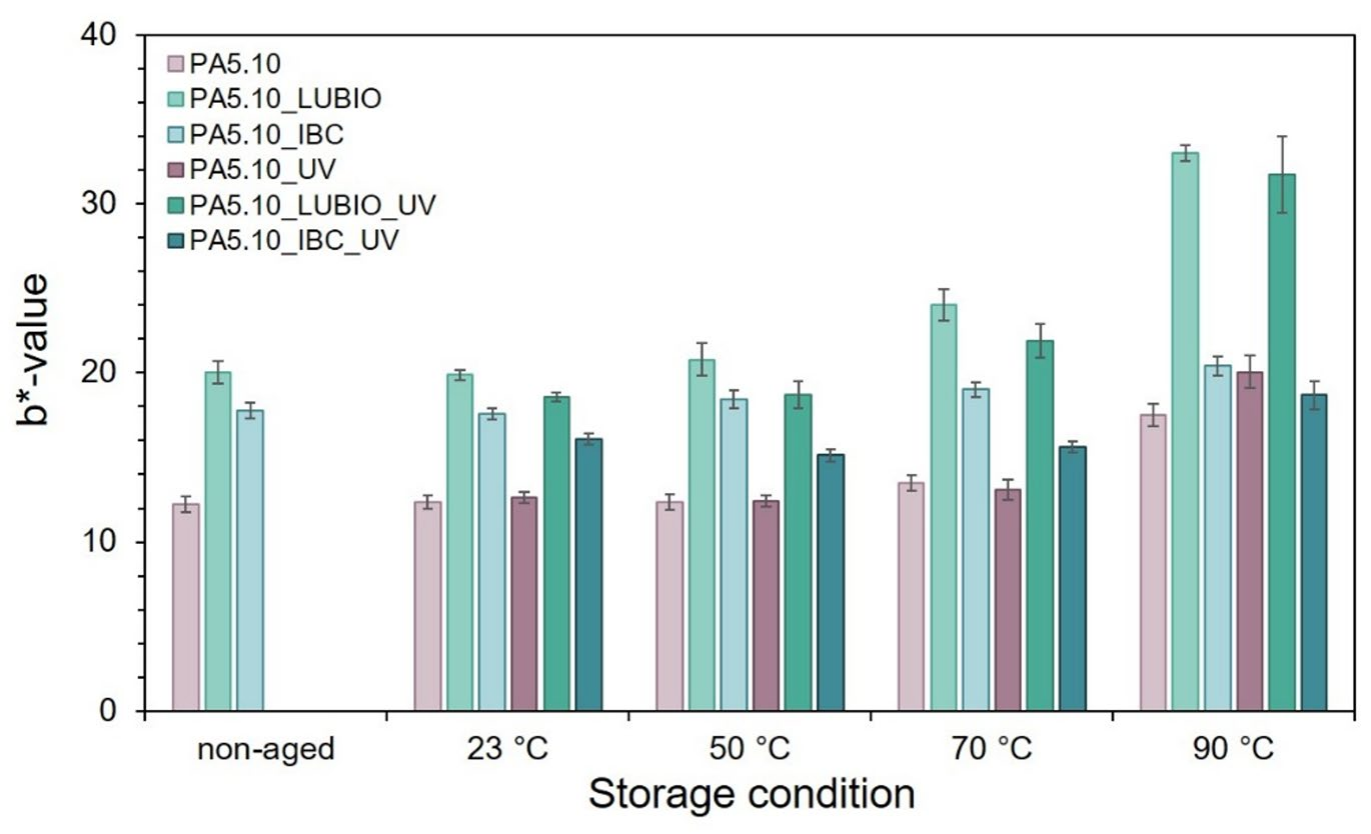
Quantification of yellowing in the three non-reinforced batches using b*-values from the CIELAB color space, presented as column charts over the storage conditions as well as the non-aged reference. The results correspond to the visual impressions shown in Fig. 8.

To assess the optical degradation behavior and evaluate the extent of discoloration, both qualitative (Fig. 8) and quantitative (Fig. 9) color analyses were carried out on the three non-reinforced PA5.10 batches. Yellowing as well as darkening are common visual indicators of polymer degradation, typically associated with thermo- and photo-oxidative aging processes. These effects appear due to chemical changes within the polymer matrix, including molecular chain splitting processes, cross-linking, and the formation of conjugated double bonds and chromophore oxidation products, such as carbonyl or amine-containing groups, which can also be seen in the FTIR spectrum in Fig. 4^62^. These degradation by-products absorb light in the visible spectrum and shift the material’s perceived color toward yellow or brown.

Pictures in Fig. 8 allow for a direct visual comparison of discoloration effects and help identify qualitative trends in surface appearance. Representative images were taken before (non-aged) and after exposure to the thermo- and photo-oxidative storage conditions. In general, increased yellowing of the neat PA5.10 was observed with elevated storage temperatures, especially under UV exposure, which is consistent with the known sensitivity of aliphatic polyamides to photo-oxidative degradation^63^. Furthermore, *Gijsman et al.* also observed, that the yellowing rate of polyamides accelerates in the presence of moisture, which can also be seen in Fig. 1 (a)^19^. Among the tested non-reinforced batches, PA5.10_LUBIO exhibited the highest degree of yellowing, both in non-aged and in stored condition. While UV absorbers are intended to protect the polymers by converting UV radiation into heat, this localized temperature increase can accelerate thermo-oxidative degradation processes. As a result, yellowing may still occur despite the presence of stabilizers, particularly under high UV exposure. Additionally, some UV absorbers or their degradation products may contribute to discoloration due to the formation of chromophore by-products over time^37,64^. In contrast, the PA5.10_IBC batch shows the least discoloration, underscoring the efficiency of its combined UV stabilization system.

To quantify these discolorations, the corresponding b*-values from the CIELAB color space were determined. The b*-value reflects the position of a color along the blue-yellow axis and increases with progressive yellowing. The results are presented in Fig. 9 for all tested conditions and complement the visual observations. The column charts show a clear correlation between increased storage temperature and b*-value, indicating accelerated discoloration at higher temperatures^21,54^. Additionally, UV exposure further intensifies yellowing, particularly in the neat PA5.10 and LUBIO-stabilized batches.

**Fig. 10:**
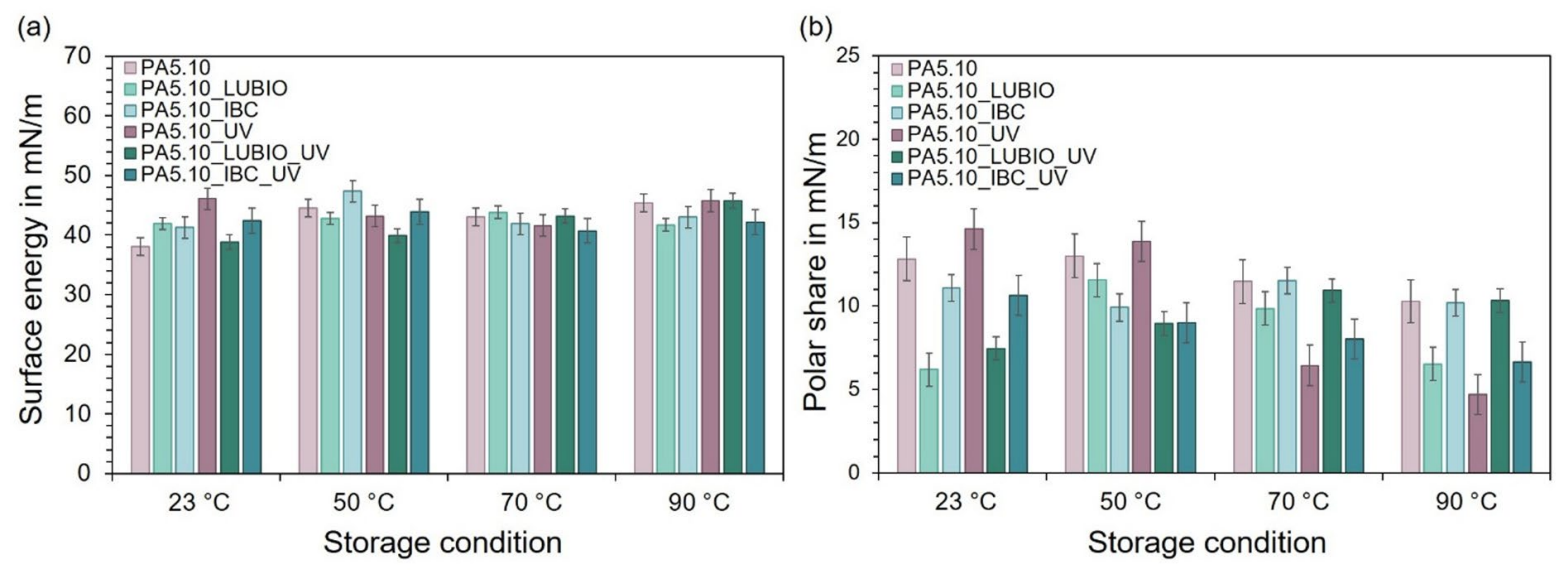
The column charts display the results of the contact angle measurements of the three non-reinforced batches after exposure to the defined storage conditions. In (**a**), the surface energy in mN/m is shown in relation to the non-aged reference and the storage temperatures of 23 °C, 50 °C, 70 °C, and 90 °C, each at 50%rH. In (**b**), only the polar share of the surface energy can be seen.

As the surface energy of the polyamide, particularly its polar share, has a significant effect on the fiber-matrix adhesion and is strongly influenced by radiation in general, contact angle measurements were carried out. Figure 10 presents the results of the contact angle measurements conducted on the three non-reinforced PA5.10 batches. In Fig. 10 (a), the total surface energy in mN/m is shown in relative to the non-aged reference and for the type 1 A test specimens stored at the thermo- and photo-oxidative aging conditions. Across all batches and storage temperatures, the total surface energy remains quite constant, suggesting that the overall wettability is only marginally influenced by thermo- or photo-oxidative degradation. Furthermore, there is no difference due to the UV stabilization visible. However, this apparent stability masks important changes on the specimen’s surface. For this reason, Fig. 10 (b) displays the isolated polar share of the surface energy, which reflects the presence of polar functional groups at the surface of the polyamide. Here, a pronounced decrease can be observed, particularly in the UV exposed neat PA5.10 specimens from 70 °C. This trend indicates a loss of the surface polarity, which can be attributed to photo-oxidative molecular chain splitting processes that lead to a breakdown or a transformation of polar groups such as amide or hydroxyl groups^65–68^. At lower thermal exposures up to 50 °C, a slight increase in polarity is observed in the PA5.10_LUBIO batch. This is likely due to cross-linking reactions or secondary crystallization processes occurring near T_g_ of PA5.10, due to annealing processes, which promote molecular reorganization and expose new polar groups on the surface^62^. In addition, a strong decrease in the polarity of the PA5.10_IBC specimens can also be observed in Fig. 10 (b). Since the IBC stabilization prevented the degradation due to thermo- and photo-oxidative processes, as it is visible in the previous measurements, this is an uncommon result. This might be because the HALS effectively suppress the formation of polar oxidation products by scavenging radicals early in the degradation process. This results in fewer polar functional groups at the surface, even though the overall material degradation is low^22,35^.

**Fig. 11:**
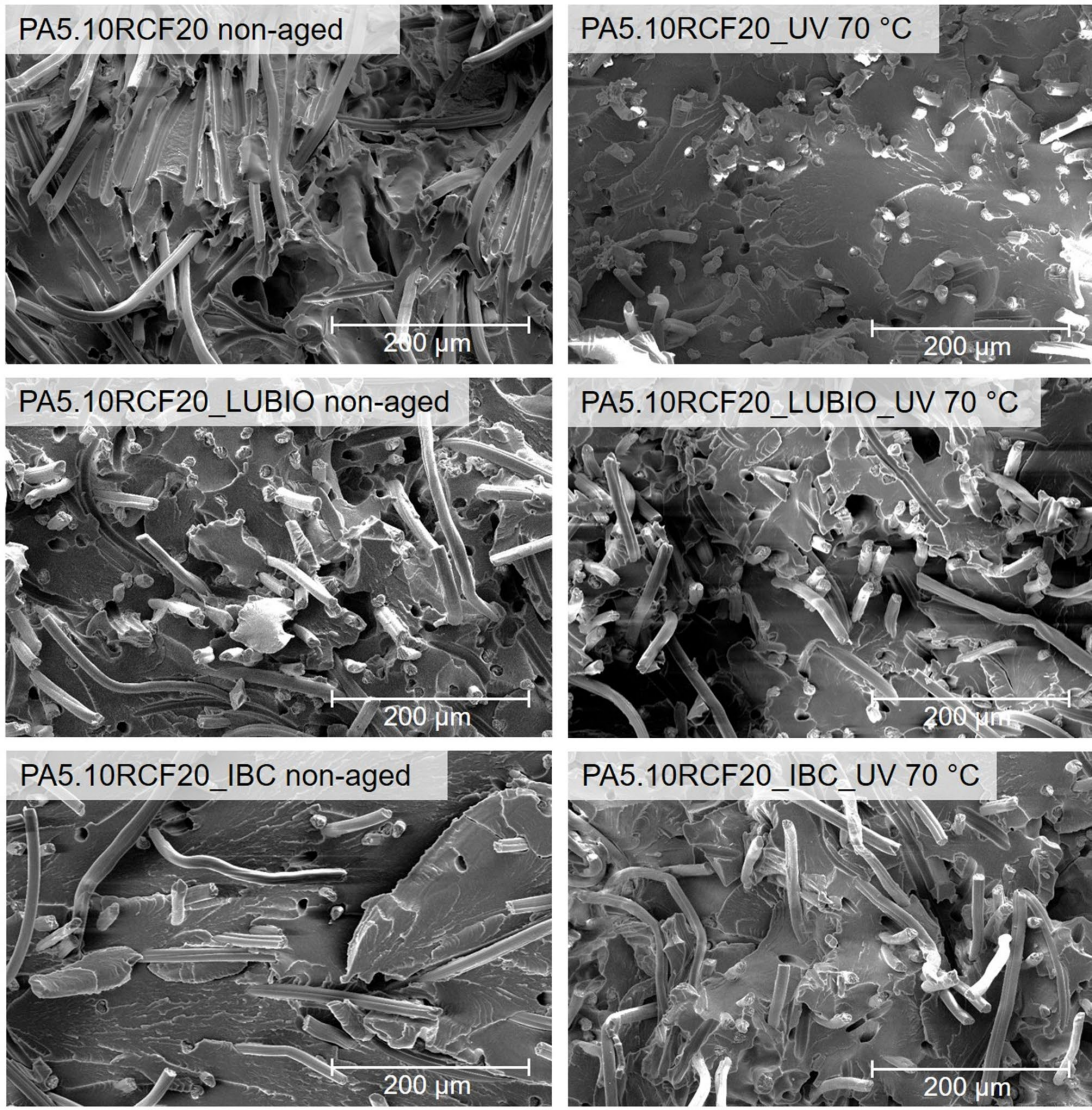
SEM images of the three RCF-reinforced PA5.10 batches in the non-aged condition and after UV exposure at 70 °C can be seen.

Using SEM images, the fiber-matrix adhesion can be qualitatively assessed. Moreover, changes of the PA5.10 matrix itself as well as the fiber pull-out and fiber rupture behavior can be observed^3^. In Fig. 11, SEM images of the fracture surfaces of the three RCF-reinforced PA5.10 batches, both in non-aged condition and after the storage under UV exposure at 70 °C, can be seen. This storage condition was selected based on the results of the previous tensile strength measurements in Fig. 5 (b), which revealed the most pronounced differences in mechanical performance among the neat PA5.10RCF20 and the UV-stabilized batches. In non-aged condition, all three batches show extensive fiber pull-outs, which indicate a limited fiber–matrix adhesion^3^. However, in non-aged condition, the PA5.10RCF20_LUBIO composite displays more frequent fiber breakage, in comparison to the other two, suggesting a stronger fiber-matrix interface that increases an effective stress transfer under mechanical stress^25,69^. After the UV exposed storage at 70 °C, microstructural differences in between the neat and UV-stabilized batches become apparent. The neat PA5.10RCF20 specimen predominantly shows fiber ruptures on the specimens fracture surface, indicating a brittle fracture behavior and a significant degradation of the PA5.10^11^. This aligns with earlier observations of a reduced elongation at break in Fig. 6 (a) and a strongly decreased notched impact strength, compared to the stabilized batches in Fig. 7. In contrast, the PA5.10RCF20_IBC as well as the PA5.10RCF20_LUBIO specimens retain long RCF pull-outs and less embrittlement. The predominance of fiber pull-out in the stabilized test specimens, even after UV aging at 70 °C, indicates a reduced level of matrix and fiber degradation. This can be attributed to the radical-scavenging mechanism of the UV stabilizers, which effectively suppress photo-chemical and thermo-oxidative processes, thereby preserving fiber integrity and maintaining a more ductile failure mechanism^22,35–37^.

### Rheological analysis

**Fig. 12:**
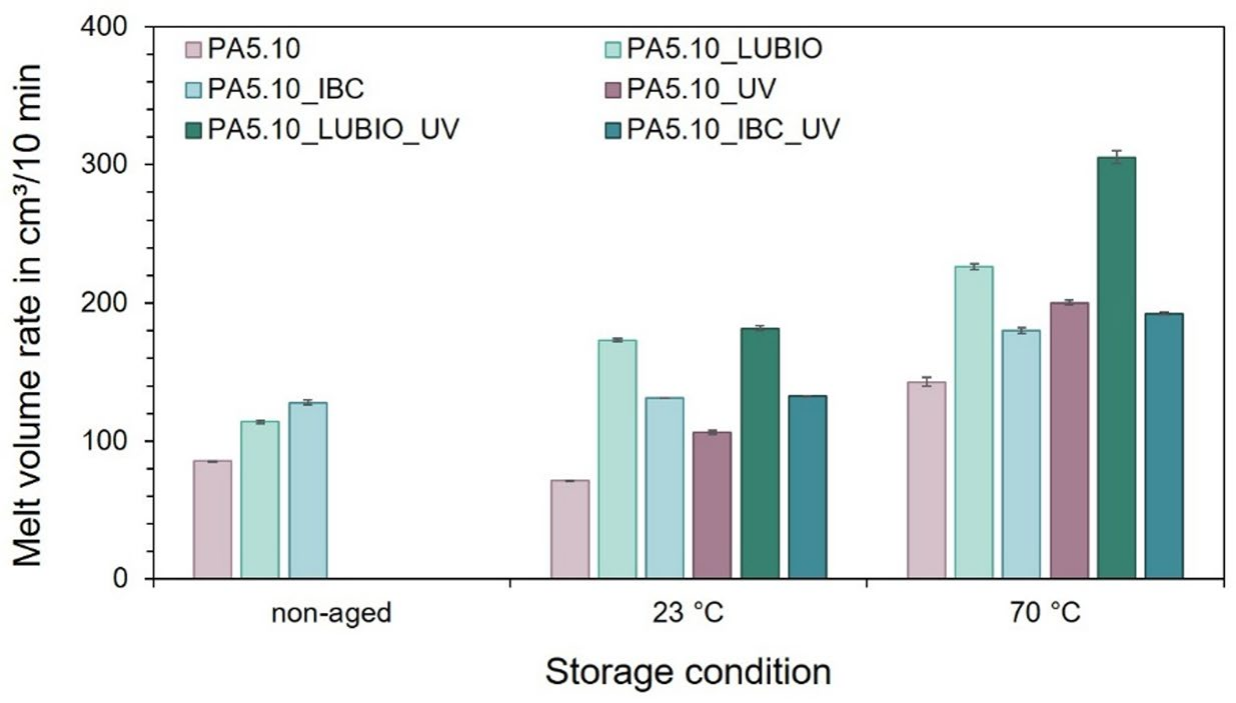
Melt volume rate (MVR) of the three non-reinforced PA5.10 batches under non-aged conditions, and after storage at 23 °C and 70 °C. The UV-stabilized batches show higher initial MVR values in the non-aged condition.

Lastly, the melt volume rate, or more specifically, the change in viscosity due to the thermo- and photo-oxidative storage conditions was investigated. Figure 12 displays the MVR in cm³/10 min of the three non-reinforced PA5.10 batches under non-aged conditions as well as after storage at 23 °C and 70 °C, both with and without UV exposure. The 70 °C condition was selected based on the tensile strength results in Fig. 5, which revealed the most significant degradation effects across the investigated batches, similar to the selection of SEM images taken. Even in the non-aged state, the UV-stabilized batches exhibit significantly higher MVR values compared to the neat PA5.10. This difference might result from variations in molecular weight distribution, possibly introduced through processing aids or the stabilizers within the additive masterbatches.

Subsequent to the storage at 70 °C, a significant increase in MVR is observed, particularly under combined thermal environment and UV exposure. This observation reflects a decrease in melt viscosity, which can be attributed to the molecular chain splitting processes caused by the synergistic effects of the thermo-oxidative and photo-oxidative degradation as well as to moisture absorption^70^. The most pronounced increase in MVR occurs in the PA5.10_LUBIO batch, indicating a higher susceptibility to molecular chain splitting processes despite the presence of the UV absorber. However, although the PA5.10_LUBIO batch shows the highest MVR increase after UV exposure, this effect is not reflected in a strong increase in crystallinity or the highest decrease tensile properties. This discrepancy suggests that the degradation mainly affects low-molecular-weight fractions or amorphous chain segments, which influence melt flow but not the stiffness and strength of the PA5.10 in the solid state. The measurement of the MVR therefore acts as a more sensitive indicator of early-stage molecular degradation, which might not yet compromise the polyamide’s structural integrity^71^. Furthermore, it supports the previously observed possible molecular chain splitting processes due to thermo- and photo-oxidative degradation of the other batches, which could be seen in the FTIR spectra, in the increase in crystallinity and in the decreasing mechanical properties, especially of the non-reinforced PA5.10.

### Structure-property analysis of crystallinity and moisture content

**Fig. 13:**
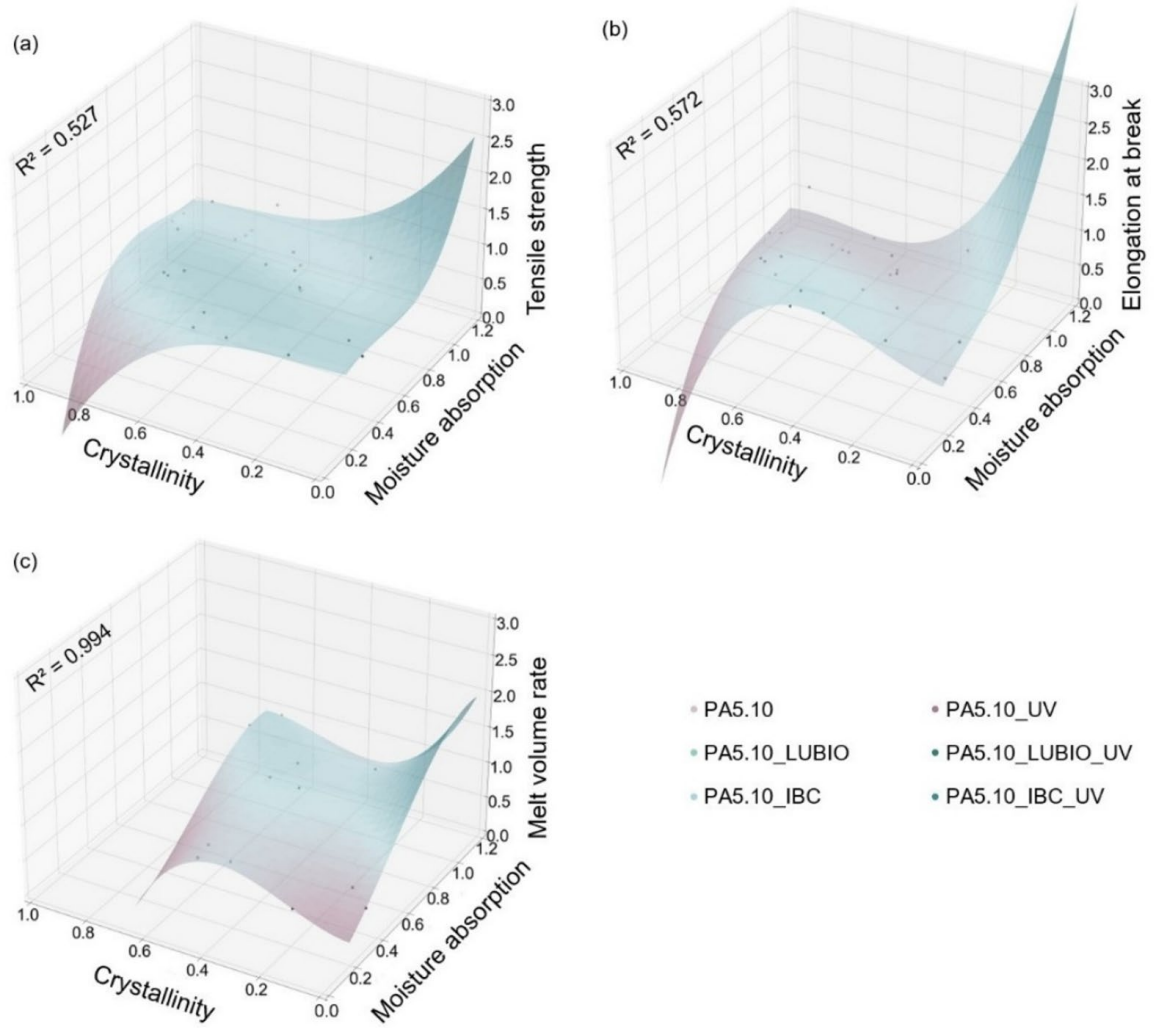
Surface plots illustrating the correlation between the moisture absorption on the x-axis, the degree in crystallinity on the y-axis and the values of the tensile strength (**a**), elongation at break (**b**) and melt volume rate (**c**) on the z-axis. All experimental data points are normalized in a range from 0–1, fitted using third-degree polynomial regression and represent the average results of all neat and stabilized non-reinforced PA5.10 specimens. The coefficient of determination R^2^ is indicated for each fit.

The degree of crystallinity is known as a critical factor influencing the properties of semi-crystalline polymers. Changes in crystallinity as a result of degradation processes can significantly affect the polyamide’s strength, ductility as well as the moisture absorption behavior and is highly dependent of the molecular chain lengths^72^. Previous studies have shown that degradation processes alter the crystalline and amorphous phases, thereby impacting the polymer’s properties^13,15,70^. Furthermore, the moisture content has a strong impact on the structural-property relations of polyamides. A high moisture content results in plasticization processes due to the incorporation and adsorption of water molecules, which lead to an increase in elongation at break and a decrease in stiffness and melt viscosity^28,73^. In addition, high moisture contents in the polyamide further accelerate hydrolytic degradation especially at temperatures above T_g_^13,14,29^. By analyzing the results of the tensile strength, elongation at break and melt volume rate of the non-reinforced PA5.10, in Fig. 13, the correlation with the increasing degree of crystallinity and moisture content, due to the thermo- and photo-oxidative degradation can be seen. Using polynomial regression, relations can be identified between the independent variables (storage conditions, stabilization), the resulting dependent output variables (tensile strength, elongation at break, melt volume rate), and the latent factors crystallinity and moisture content. The coefficients of the 3rd-degree polynomial regression, as well as the R^2^ value, can be seen in Table 3.

**Table 3:** Coefficients of the linear polynomial regression of the average results of all neat and stabilized non-reinforced PA5.10 specimens after storage at thermo- and photo-oxidative conditions for tensile strength, elongation at break and melt volume rate. All experimental data points are normalized in a range from 0–1, fitted using third-degree polynomial regression. The coefficient of determination R^2^ is indicated for each fit.

PA5.10			
	Tensile strength	Elongation at break	Melt volume rate
a	0.567	0.232	0.027
b1	−0.801	1.734	2.195
b2	3.315	2.564	0.658
c1	0.166	2.77	4.854
c2	3.941	−5.937	−10.55
c3	−7.533	−3.943	3.818
d1	−3.285	−8.857	−11.058
d2	6.66	15.753	11.494
d3	−7.74	−8.212	0.943
d4	5.364	4.099	−2.503
R^2^	0.527	0.572	0.994

The R^2^ values for tensile strength and elongation at break are 0.527 and 0.572, whereas the R^2^ for MVR is at 0.994. The higher R^2^ for MVR reflects a closer fit of the polynomial regression to the experimental data for this property. The lower R^2^ values for tensile strength and elongation at break indicate that the correlation with crystallinity and moisture content is weaker, which is expected given the more complex and multifactorial nature of mechanical properties compared to MVR. While a higher-degree polynomial could improve the R^2^ values, this would risk overfitting and reduce the generalizability of the model; therefore, a third-degree polynomial was chosen as a compromise, providing a reasonable description of the trends while avoiding excessive fitting. In Table 3, the intercept a is the expected value of y when the values of the crystallinity and moisture content are zero which provides a baseline for predictions. The linear coefficients b_1_ and b_2_ indicate the average change in y for a one-unit increase in crystallinity (b_1_) or moisture content (b_2_), while the other variable remains constant. The quadratic and cubic coefficients c_1_ to c_3_ and d_1_ to d_4_ show the non-linear effect on y as the crystallinity or moisture absorption increases.

Figure 13 (a) shows the correlation between crystallinity, moisture content and tensile strength. This shows a decreasing trend with increasing crystallinity. This is initially counter-intuitive, as higher degrees of crystallinity are usually followed by higher strength and stiffness^72^. The increase in crystallinity observed is not attributed to conventional process-induced factors such as slower cooling and the resulting chain rearrangement, but rather to molecular chain splitting processes and the accelerated reordering of the resulting shorter chain fragments. These shortened chains are more capable of organizing into crystalline structures at temperatures above T_g_, leading to a higher overall degree of crystallinity^74^. Moreover, the relative degree of crystallinity further increases due to the degradation of the amorphous regions^13,14^. Since the amorphous regions in the polyamide absorb the moisture, a higher degree of crystallinity should also lead to a decreased moisture absorption and therefore a lower tensile strength. As opposed to this, the thermo- and photo-oxidative storage allowed the polyamide to absorb moisture at an accelerated rate, thus showing an interaction between the plasticization of the matrix material through moisture absorption and embrittlement due to molecular chain splitting processes, which can also be seen in the high tensile strength in Fig. 13 (a)^25^.

When examining the elongation at break in Fig. 13 (b), the trends correspond to the literature, as the increased crystallinity is accompanied by a reduced elongation at break^70^. In this case, it does not make a difference whether the increase in crystallinity was caused by altered process conditions during production of the type 1 A test specimens or thermal degradation. In contrast to Fig. 13 (a), however, it can be seen that the decrease in elongation at break is significantly larger than the drop in tensile strength. Shorter molecular chains, microcracks and structural defects formed during aging can act as stress concentrators, promoting premature failure under tensile loading and sharply reducing the polyamide’s ductility, while tensile strength is often less severely affected^16^. The increase in elongation at break at higher moisture contents is due to the plasticization of the material and is in accordance with the literature, as well^13,28^. The correlated results of the MVR can be seen on Fig. 13 (c). The ascending trend for the MVR with increasing crystallinity results from molecular chain splitting processes due to the thermo- and photo-oxidative degradation as well as to moisture absorption, which reduce the viscosity of the polyamide^70^.

## Conclusions

This study investigated the synergistic effects of thermo- and photo-oxidative degradation on neat and RCF-reinforced PA5.10 composites stabilized with two UV stabilizer systems, AddWorks® IBC760 and LUBIO® UV 16. The combined thermal, mechanical, optical, and rheological analyses provided a comprehensive analysis of how temperature, UV radiation, and moisture interact to affect the stability of the PA5.10, revealing consistent trends across all methods. Structural changes detected by FTIR correlated well with reductions in tensile strength and elongation at break, as well as with increases in MVR, supporting the observed synergistic degradation behavior. Minor deviations observed in some RCF-reinforced composites, such as temporarily increased ductility due to moisture-related plasticization, are consistent with the hygroscopic nature of the RCF and are discussed in the results section.

Neat PA5.10 showed pronounced degradation under elevated temperature and UV exposure, manifested by molecular chain splitting processes, embrittlement, and strong yellowing. FTIR spectroscopy indicated structural alterations in the carbonyl region (at 1700 cm^−1^), consistent with oxidation and molecular rearrangements. These changes correlated with reduced elongation at break and increased MVR, confirming a loss in molecular weight. RCF-reinforcement modified the degradation response. While the RCF introduced moisture-related plasticization that initially increased ductility, prolonged UV exposure led to fiber embrittlement and decreased tensile strength. SEM observations supported this trend, showing a shift from ductile fiber pull-out in non-aged composites to brittle fiber fracture after UV exposure. The stabilizer systems displayed markedly different efficiencies. The IBC-stabilizer provided the most effective and consistent protection across all test conditions, maintaining mechanical performance, color stability, and molecular integrity by efficiently suppressing radical reactions. In contrast, the LUBIO offered limited stabilization, especially under combined UV and thermal stress, suggesting its lower efficiency in preventing secondary degradation once the polyamide backbone is damaged.

Overall, degradation in PA5.10, both neat and RCF-reinforced, was significantly stronger under UV exposure than under thermal aging alone, with increasing temperature accelerating photo-oxidative reactions. The observed effects under combined UV and thermal aging were often more pronounced than the sum of the individual UV and thermal degradations, indicating a synergistic interaction between temperature and photo-chemical oxidation. This highlights that considering thermal or UV aging separately may underestimate the actual degradation in in-use conditions. Importantly, this study demonstrates for the first time how RCF influence the degradation behavior of PA5.10 under simultaneous UV and thermal stress, providing new insights into fiber-matrix interactions and synergistic degradation mechanisms in sustainable composites. The results highlight that integrating an efficient stabilization system, particularly IBC, is crucial to ensuring the long-term durability of bio-based PA5.10. These findings underline the potential of UV-stabilized PA5.10 composites as sustainable alternatives to petrochemical polyamides for applications requiring prolonged resistance to thermo- and photo-oxidative environments.

## Data Availability

The datasets generated and/or analyzed in the present study are available in repository of the University of Kassel DaKS (10.48662/daks-275) on request.

## References

[1] Sanjay, M. R. et al. Characterization and properties of natural fiber polymer composites: A comprehensive review. *J. Clean. Prod.* **172**, 566–581. 10.1016/j.jclepro.2017.10.101] (2018).

[2] Väisänen, T., Das, O. & Tomppo, L. A review on new bio-based constituents for natural fiber-polymer composites. *J. Clean. Prod.* **149**, 582–596. 10.1016/j.jclepro.2017.02.132] (2017).

[3] Zarges, J-C., Minkley, D., Feldmann, M. & Heim, H-P. Fracture toughness of injection molded, man-made cellulose fiber reinforced polypropylene. *Compos. Part A: Appl. Sci. Manufac.* **98**, 147–158. 10.1016/j.compositesa.2017.03.022] (2017).

[4] Sälzer, P., Feldmann, M. & Heim, H. P. Wood-Polypropylene Composites: Influence of Processing on the Particle Shape and Size in Correlation with the Mechanical Properties Using Dynamic Image Analysis. *Int. Polym. Proc.* **33** (5), 677–687. 10.3139/217.3446] (2018).

[5] Praveenkumara, J. et al. Recent developments and challenges in natural fiber composites: A review. *Polym. Compos.* **43** (5), 2545–2561. 10.1002/pc.26619] (2022).

[6] Goetjes, V., Zarges, J-C. & Heim, H-P. Differentiation between Hydrolytic and Thermo-Oxidative Degradation of Poly(lactic acid) and Poly(lactic acid)/Starch Composites in Warm and Humid Environments. *Materials* **17** (15), 3683. 10.3390/ma17153683] (2024). 39124345 10.3390/ma17153683 PMC11313141

[7] Goetjes, V., Zarges, J-C. & Heim, H-P. Resistance of poly(lactic acid)/starch composites to weathering effects. *J. Appl. Polym. Sci.*10.1002/app.54768] (2023).

[8] Falkenreck, C. K., Korthals, H. S., Goetjes, V. & Heim, H. P. Textile Waste becomes plastic composites: Cotton as Fiber-Reinforcement. *Plast. Insights - Mater. - Process. - Appl.* 62–64. 10.17170/kobra-202402069529] (2023).

[9] Mejri, M., Toubal, L., Cuillière, J. C. & François, V. Hygrothermal aging effects on mechanical and fatigue behaviors of a short- natural-fiber-reinforced composite. *Int. J. Fatigue*. **108**, 96–108. 10.1016/j.ijfatigue.2017.11.004] (2018).

[10] Falkenreck, C. K., Zarges, J-C. & Heim, H-P. Hygrothermal Aging Behavior of Regenerated Cellulose Fiber-Reinforced Polyamide 5.10 Composites. *Polym. Test.* 108717. 10.1016/j.polymertesting.2025.108717] (2025).

[11] Falkenreck, C. K., Gemmeke, N., Zarges, J-C. & Heim, H-P. Influence of Accelerated Aging on the Fiber–Matrix Adhesion of Regenerated Cellulose Fiber-Reinforced Bio-Polyamide. *Polym. (Basel)*. 10.3390/polym15071606] (2023). 10.3390/polym15071606 PMC10097342 37050220

[12] Arhant, M. et al. Modelling the non Fickian water absorption in polyamide 6. *Polym. Degrad. Stab.* **133**, 404–412. 10.1016/j.polymdegradstab.2016.09.001] (2016).

[13] Deshoulles, Q. et al. Origin of embrittlement in Polyamide 6 induced by chemical degradations: mechanisms and governing factors. *Polym. Degrad. Stab.* 191. 10.1016/j.polymdegradstab.2021.109657] (2021).

[14] Jacques, B., Werth, M., Merda, I., Thominette, F. & Verdu, J. Hydrolytic ageing of polyamide 11. 1. Hydrolysis kinetics in water. *Polymer* **43**, 6439–6447 (2002).

[15] Cerruti, P. & Carfagna, C. Thermal-oxidative degradation of polyamide 6,6 containing metal salts. *Polym. Degrad. Stab.* **95** (12), 2405–2412. 10.1016/j.polymdegradstab.2010.08.014] (2010).

[16] Shu, Y., Ye, L. & Yang, T. Study on the long-term thermal-oxidative aging behavior of polyamide 6. *J. Appl. Polym. Sci.* **110** (2), 945–957. 10.1002/app.28647] (2008).

[17] Shi, K., Ye, L. & Li, G. Thermal oxidative aging behavior and stabilizing mechanism of highly oriented polyamide 6. *J. Therm. Anal. Calorim.* **126** (2), 795–805. 10.1007/s10973-016-5523-6] (2016).

[18] Richaud, E. et al. Auto-oxidation of aliphatic polyamides. *Polym. Degrad. Stab.* **98** (9), 1929–1939. 10.1016/j.polymdegradstab.2013.04.012] (2013).

[19] Gijsman, P. Review on the thermo-oxidative degradation of polymers during processing and in service. e-Polymers. ; (65). (2008).

[20] Gijsman, P., Hensen, G. & Mak, M. Thermal initiation of the oxidation of thermoplastic polymers (Polyamides, Polyesters and UHMwPE). *Polym. Degrad. Stab.* **183**, 109452. 10.1016/j.polymdegradstab.2020.109452] (2021).

[21] Hurtado Macias, A. et al., *Comparative study between UVB 313 nm, UVC 254 nm, and far UVC 222 nm light on the aging of polyamide 66. Heliyon. ; 10(20): e39415 [*10.1016/j.heliyon.2024.e*39415][PMID: 39506960]* (2024). 10.1016/j.heliyon.2024.e39415 PMC11538952 39506960

[22] Step, E. N., Turro, N. J., Gande, M. E. & // Klemchuk. Mechanism of Polymer Stabilization by Hindered-Amine Light Stabilizers (HALS). Model Investigations of the Interaction of Peroxy Radicals with HALS Amines and Amino Ethers. *Macromolecules* **27**, 2529–2539. 10.1021/ma00087a022] (1994).

[23] Gijsman, P., Meijers, G. & Vitarelli, G. Comparison of the UV-degradation chemistry of polypropylene, polyethylene, polyamide 6 and polybutylene terephthalate. *Polym. Degrad. Stab.* **65**, 433–441 (1999).

[24] Carroccio, S., Puglisi, C. & Montaudo, G. New Vistas in the Photo-Oxidation of Nylon 6. *Macromolecules* **36** (20), 7499–7507. 10.1021/ma0344137] (2003).

[25] Falkenreck, C. K., Zarges, J-C., Heim, H-P., Seitz, M. & Bonten, C. Degradation of regenerated cellulose fiber-reinforced bio-polyamide in hydrothermal environment. *Compos. Part A: Appl. Sci. Manufac.* **188**, 108584. 10.1016/j.compositesa.2024.108584] (2025).

[26] Akindoyo, J. O. et al. Oxidative induction and performance of oil palm fiber reinforced polypropylene composites – Effects of coupling agent and UV stabilizer. *Compos. Part A: Appl. Sci. Manufac.* **125**, 105577. 10.1016/j.compositesa.2019.105577] (2019).

[27] Elsevier (ed) *Durability and Life Prediction in Biocomposites, Fibre-Reinforced Composites and Hybrid Composites* (Elsevier, 2019).

[28] Deshoulles, Q. et al. Chemical coupling between oxidation and hydrolysis in polyamide 6. *Polym. Degrad. Stab.* 197. 10.1016/j.polymdegradstab.2022.109851] (2022).

[29] Hocker, S., Rhudy, A. K., Ginsburg, G. & Kranbuehl, D. E. Polyamide hydrolysis accelerated by small weak organic acids. *Polymer* **55** (20), 5057–5064. 10.1016/j.polymer.2014.08.010] (2014).

[30] Akro-Plastic. *Technical Datasheet AKROMID® NEXT 5.10 3 EXP natur* (Akro-Plastic, 2022).

[31] Kind, S. et al. From zero to hero - production of bio-based nylon from renewable resources using engineered Corynebacterium glutamicum. *Metab. Eng.* **25**, 113–123. 10.1016/j.ymben.2014.05 (2014). .007][PMID: 24831706]. 24831706 10.1016/j.ymben.2014.05.007

[32] Kind, S. & Wittmann, C. Bio-based production of the platform chemical 1,5-diaminopentane. *Appl. Microbiol. Biotechnol.* **91** (5), 1287–1296. 10.1007/s00253-011-3457-2] (2011). 21761208 10.1007/s00253-011-3457-2

[33] Woodings, C. *Regenerated cellulose fibres* (Woodhead Publ, 2001).

[34] Lang, D. *TDS Cordenka Rayon Chopped Fibers* (Cordenka GmbH & Co. KG, 2021).

[35] Clariant, S. E. Technical Data Sheet - AddWorks^®^ IBC 760; Nov 17. (2020).

[36] Schäfer Additivsysteme GmbH. Technical Data Sheet - LUBIO UV 16, V1.0.0. Apr 5. (2017).

[37] Nomura, K. & Terwilliger, P. Application of antioxidant and ultraviolet absorber into HDPE: Enhanced resistance to UV irradiation. *Special Matrices*. **7** (1), 1–19. 10.1515/spma-2019-0001] (2019).

[38] Acierno, S. & van Puyvelde, P. Rheological behavior of polyamide 11 with varying initial moisture content. *J. Appl. Polym. Sci.* **97** (2), 666–670. 10.1002/app.21810] (2005).

[39] Feldmann, M. The effects of the injection moulding temperature on the mechanical properties and morphology of polypropylene man-made cellulose fibre composites. *Compos. Part A: Appl. Sci. Manufac.* **87**, 146–152. 10.1016/j.compositesa.2016.04.022] (2016).

[40] Beuth Verlag. DIN EN ISO 291:2008.

[41] Millot, C., Fillot, L-A., Lame, O., Sotta, P. & Seguela, R. Assessment of polyamide-6 crystallinity by DSC. *J. Therm. Anal. Calorim.* **122** (1), 307–314. 10.1007/s10973-015-4670-5] (2015).

[42] Ehrenstein, G. W. Thermische Analyse: Kapitel 3 - Dynamische Differenzkalorimetrie. Carl Hanser.

[43] Pedregosa, F. et al. Scikit-learn: Machine Learning in Python. *J. Mach. Learn. Res.* **12**, 2825–2830. 10.5555/1953048.2078195 (2011).

[44] Harris, C. R. et al. Array programming with NumPy. *Nature* **585** (7825), 357–362. 10.1038/s41586-020- (2020). 2649-2][PMID: 32939066]. 32939066 10.1038/s41586-020-2649-2 PMC7759461

[45] Hunter, J. D. & Matplotlib A 2D Graphics Environment. COMPUTING IN SCIENCE. *Eng.* 90–95. 10.1109/MCSE.2007.55] (2007).

[46] McKinney, W. & IN SCIENCE CONF. Data Structures for Statistical Computing in Python. PROC. OF THE 9th PYTHON : 56–61 [10.25080/Majora-92bf1922-00a]

[47] Draper, N. R. & Smith, H. *Applied Regression Analysis* (John Wiley & Sons Inc, 1998).

[48] Ostertagová, E. Modelling using Polynomial Regression. *Procedia Eng.* **48**, 500–506. 10.1016/j.proeng.2012.09.545] (2012).

[49] Falkenreck, C. K., Zarges, J-C. & Heim, H-P. Degradation pathways and chemical stability of regenerated cellulose fiber-reinforced bio-polyamide 5.10 composites under acidic and alkaline conditions. *Sci. Rep.* **15** (1). 10.1038/s41598-025-23295-2] (2025). 10.1038/s41598-025-23295-2 PMC12511547 41068290

[50] Cundiff, K. N., Rodriguez, A. K. & Benzerga, A. A. Mechanical behavior of polyamide-6 after combined photo-oxidative and hygrothermal aging. *Colloid Polym. Sci.* **302** (4), 609–622. 10.1007/s00396-023-05218-7] (2024).

[51] Sang, L., Wang, C., Wang, Y. & Hou, W. Effects of hydrothermal aging on moisture absorption and property prediction of short carbon fiber reinforced polyamide 6 composites. *Compos. Part. B: Eng.* **153**, 306–314. 10.1016/j.compositesb.2018.08.138] (2018).

[52] Li, X., Wang, L., Wang, D., Müller, A. J. & Dong, X. Competition between Chain Extension and Crosslinking in Polyamide 1012 during High-Temperature Thermal Treatments as Revealed by Successive Self-Nucleation and Annealing Fractionation. *Macromolecules* **54** (16), 7552–7563. 10.1021/acs.macromol.1c01252] (2021).

[53] Li, X. et al. Effect of Initial Molecular Weight on the Structural Evolution of Polyamide 1012 during High-Temperature Thermal Treatments as Revealed by Successive Self-Nucleation and Annealing. *Macromolecules* **55** (17), 7674–7682. 10.1021/acs.macromol.2c01165] (2022).

[54] Shackleford, A. S., Williams, R. J., Brown, R., Wingham, J. R. & Majewski, C. Degradation of Laser Sintered polyamide 12 parts due to accelerated exposure to ultraviolet radiation. *Additive Manuf.* **46**, 102132. 10.1016/j.addma.2021.102132] (2021).

[55] Katja Klump. Stabilisierung von Polyamiden bezüglich der Lagerung bei hohen Temperaturen. Dissertation, Fachbereich Chemie, Technischen Universität Darmstadt (2021).

[56] Do, C. H., Pearce, E. M. & Bulkin, B. J. FT–IR spectroscopic study on the thermal and thermal oxidative degradation of nylons. *J. Polym. Science: Part. A: Polym. Chem.* **25**, 2409–2424 (1987).

[57] Liquori, A. M., Mele, A. & Carelli, V. Ultraviolet absorption spectra of polyamides. *J. Polym. Sci.* **10** (5), 510–512. 10.1002/pol.1953.120100510] (1953).

[58] Marek, A. A. & Verney, V. Rheological behavior of polyolefins during UV irradiation at high temperature as a coupled degradative process. *Eur. Polymer J.* **72**, 1–11. 10.1016/j.eurpolymj.2015.09.003] (2015).

[59] Falkenreck, C. K., Liehr, A., Zarges, J-C., Heim, H-P. & Niendorf, T. Correlation of the crystalline structure, moisture content, texture and aging-induced degradation of polyamide 5.10 using wide-angle x-ray scattering analysis. *Polym. Degrad. Stab.* 111620. 10.1016/j.polymdegradstab.2025.111620] (2025).

[60] Thomason, J. L., Ali, J. Z. & Anderson, J. The thermo-mechanical performance of glass-fibre reinforced polyamide 66 during glycol–water hydrolysis conditioning. *Compos. Part A: Appl. Sci. Manufac.* **41** (7), 820–826. 10.1016/j.compositesa.2010.02.006] (2010).

[61] Deshoulles, Q. et al. Modelling pure polyamide 6 hydrolysis: Influence of water content in the amorphous phase. *Polym. Degrad. Stab.* **183**, 109435. 10.1016/j.polymdegradstab.2020.109435] (2021).

[62] Andrady, A. L., Hamid, S. H., Hu, X. & Torikai, A. Effects of increased solar ultraviolet radiation on materials. *J. Photochem. Photobiology*. **46**, 96–103 (1998). 10.1016/s1011-1344(98)00188-2 9894353

[63] Ching, Y. C. et al., *Effects of high temperature and ultraviolet radiation on polymer composites. In:; 407–426 .*

[64] El-HitiGA et al. Modifications of Polymers through the Addition of Ultraviolet Absorbers to Reduce the Aging Effect of Accelerated and Natural Irradiation. *Polym. (Basel)*. **14** (1). 10.3390/polym14010020 (2021). PMID: 35012042. 10.3390/polym14010020 PMC8747282 35012042

[65] Beake, B. D., Lingb, J. S. G. & Leggett, G. J. Correlation of friction, adhesion, wettability and surface chemistry after argon plasma treatment of poly(ethylene terephthalate). *J. Mater. Chem.* (8), 2845–2854. 10.1039/A807261B] (1998).

[66] Chen, N., Maeda, N., Tirrell, M. & Israelachvili, J. Adhesion and Friction of Polymer Surfaces: The Effect of Chain Ends. *Macromolecules* **38** (8), 3491–3503. 10.1021/ma047733e] (2005).

[67] Kahl, C., Gemmeke, N., Bagnucki, J. & Heim, H-P. Investigations on fiber–matrix properties of heat-treated and UV-treated regenerated cellulose fibers. *Compos. Part A: Appl. Sci. Manufac.* **152**, 106669. 10.1016/j.compositesa.2021.106669] (2022).

[68] Baldi, L. D., Iamazaki, E. T. & Atvars, T. D. Evaluation of the polarity of polyamide surfaces using the fluorescence emission of pyrene. *Dyes Pigm.* **76** (3), 669–676. 10.1016/j.dyepig.2007.01.011] (2008).

[69] Zarges, J-C., Kaufhold, C., Feldmann, M. & Heim, H-P. Single fiber pull-out test of regenerated cellulose fibers in polypropylene: An energetic evaluation. *Compos. Part A: Appl. Sci. Manufac.* **105**, 19–27. 10.1016/j.compositesa.2017.10.030] (2018).

[70] Salvi, A., Marzullo, F., Ostrowska, M. & Dotelli, G. Thermal Degradation of Glass Fibre-Reinforced Polyamide 6,6 Composites: Investigation by Accelerated Thermal Ageing. *Polym. (Basel)*. **17** (4). 10.3390/polym17040509 (2025). PMID: 40006171. 10.3390/polym17040509 PMC11859150 40006171

[71] Aldhafeeri, T., Alotaibi, M. & BarryCF Impact of Melt Processing Conditions on the Degradation of Polylactic Acid. *Polym. (Basel)*. **14** (14). 10.3390/polym14142790 (2022). PMID: 35890566. 10.3390/polym14142790 PMC9320002 35890566

[72] Yoshida, T., Nakane, T., Uchida, M. & Kaneko, Y. Mechanical modeling and testing of different polyamides considering molecular chain structure, crystallinity, and large strains. *Int. J. Solids Struct.* 239–240. 10.1016/j.ijsolstr.2021.111419] (2022).

[73] Sharma, P. et al. Moisture transport in PA6 and its influence on the mechanical properties. *Continuum Mech. Thermodyn.* **32** (2), 307–325. 10.1007/s00161-019-00815-w] (2020).

[74] Maïza, S. et al. Physicochemical and mechanical degradation of polyamide 11 induced by hydrolysis and thermal aging. *J. Appl. Polym. Sci.* **136** (23), 47628. 10.1002/app.47628] (2019).

